# An update of pathogenic variants in *ASPM*, *WDR62, CDK5RAP2*,* STIL, CENPJ,* and* CEP135* underlying autosomal recessive primary microcephaly in 32 consanguineous families from Pakistan

**DOI:** 10.1002/mgg3.1408

**Published:** 2020-07-17

**Authors:** Sajida Rasool, Jamshaid Mahmood Baig, Abubakar Moawia, Ilyas Ahmad, Maria Iqbal, Syeda Seema Waseem, Maria Asif, Uzma Abdullah, Ehtisham Ul Haq Makhdoom, Emrah Kaygusuz, Muhammad Zakaria, Shafaq Ramzan, Saif ul Haque, Asif Mir, Iram Anjum, Mehak Fiaz, Zafar Ali, Muhammad Tariq, Neelam Saba, Wajid Hussain, Birgit Budde, Saba Irshad, Angelika Anna Noegel, Stefan Höning, Shahid Mahmood Baig, Peter Nürnberg, Muhammad Sajid Hussain

**Affiliations:** ^1^ Cologne Center for Genomics (CCG) University of Cologne Cologne Germany; ^2^ Institute of Biochemistry and Biotechnology Quaid‐e‐Azam Campus University of the Punjab Lahore Pakistan; ^3^ Department of Bioinformatics & Biotechnology Faculty of Basic and Applied Sciences International Islamic University Islamabad Pakistan; ^4^ Human Molecular Genetics Laboratory Health Biotechnology Division National Institute for Biotechnology and Genetic Engineering (NIBGE) College, PIEAS Faisalabad Pakistan; ^5^ Institute for Cardiogenetics University of Luebeck Luebeck Germany; ^6^ Institute of Biochemistry I Medical Faculty University of Cologne Cologne Germany; ^7^ University Institute of Biochemistry and Biotechnology (UIBB) PMAS‐ARID Agriculture University Rawalpindi Pakistan; ^8^ Institute of Human Genetics University Medical Center Göttingen Göttingen Germany; ^9^ Nuclear Medicine, Oncology and Radiotherapy Institute (NORI) Islamabad Pakistan; ^10^ Department of Biotechnology Kinnaird College University Lahore Lahore Pakistan; ^11^ Department of Zoology University of Okara Okara Pakistan; ^12^ Center for Molecular Medicine Cologne (CMMC) University of Cologne Cologne Germany; ^13^ Bilecik Şeyh Edebali University, Molecular Biology and Genetics, Gülümbe Campus Bilecik Turkey

**Keywords:** *ASPM*, *CDK5RAP2*, *CENPJ*, *CEP135*, MCPH, *STIL*

## Abstract

**Background:**

Primary microcephaly (MCPH) is a congenital neurodevelopmental disorder manifesting as small brain and intellectual disability. It underlies isolated reduction of the cerebral cortex that is reminiscent of early hominids which makes it suitable model disease to study the hominin‐specific volumetric expansion of brain. Mutations in 25 genes have been reported to cause this disorder. Although majority of these genes were discovered in the Pakistani population, still a significant proportion of these families remains uninvestigated.

**Methods:**

We studied a cohort of 32 MCPH families from different regions of Pakistan. For disease gene identification, genome‐wide linkage analysis, Sanger sequencing, gene panel, and whole‐exome sequencing were performed.

**Results:**

By employing these techniques individually or in combination, we were able to discern relevant disease‐causing DNA variants. Collectively, 15 novel mutations were observed in five different MCPH genes; *ASPM* (10), *WDR62* (1), *CDK5RAP2* (1), *STIL* (2), and *CEP135* (1). In addition, 16 known mutations were also verified. We reviewed the literature and documented the published mutations in six MCPH genes. Intriguingly, our cohort also revealed a recurrent mutation, c.7782_7783delGA;p.(Lys2595Serfs*6), of *ASPM* reported worldwide. Drawing from this collective data, we propose two founder mutations, *ASPM*:c.9557C>G;p.(Ser3186*) and *CENPJ*:c.18delC;p.(Ser7Profs*2), in the Pakistani population.

**Conclusions:**

We discovered novel DNA variants, impairing the function of genes indispensable to build a proper functioning brain. Our study expands the mutational spectra of known MCPH genes and also provides supporting evidence to the pathogenicity of previously reported mutations. These novel DNA variants will be helpful for the clinicians and geneticists for establishing reliable diagnostic strategies for MCPH families.

## Graphical abstract

Genomic analyses of 32 Pakistani families manifesting primary microcephaly.

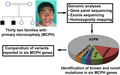

## 1 INTRODUCTION

Autosomal recessive primary microcephaly (MCPH, MIM #251200) is a rare neurodevelopmental disorder that is diagnosed by simultaneous observation of reduced head circumference (HC) of −3 SD (standard deviation)—−2 SD at birth—below the expected mean and impaired cognition. Neuroimaging of MCPH patients shows isolated cortical hypoplasia with preserved architecture and simplified gyration (Shaheen et al., 2019; Zaqout, Morris‐Rosendahl, & Kaindl, 2017). MCPH is recognized as a rare disorder, though it is highly prevalent in countries with a high rate of consanguinity, like Pakistan (1/10,000), while it is only sporadically observed (1/1,000,000) in European populations (Cox, Jackson, Bond, & Woods, 2006). The genetic etiology of MCPH is heterogeneous with mutations reported in as many as 25 different genes playing roles in diverse cellular pathways (Table S1). Among these, *ASPM* (MIM#605481) alone accounts for 68% of cases followed by 14% by *WDR62* (MIM#613583) and 8% by *MCPH1* (MIM#607117) (Zaqout et al., 2017), whereas other genes were reported only in a few families. The most frequently observed pathomechanism is abnormal spindle orientation in neural progenitor cells (NPCs), resulting in premature switching from both symmetric proliferative and asymmetric self‐renewing to symmetric consumptive division (Fei, Haffner, & Huttner, 2014; Fish, Kosodo, Enard, Paabo, & Huttner, 2006). It has been proposed that the dysfunction of the MCPH proteins either dysregulates cell cycle dynamics or increases apoptosis, both of which might disrupt mitotic neurogenesis leading to the MCPH phenotype (Cox et al., 2006; Zaqout et al., 2017).

## 2 MATERIALS AND METHODS

### 2.1 Patient consent and ethics approval

With informed consent from parent(s)/guardians, blood samples and clinical information were collected following the rules described in the Declaration of Helsinki. The study was approved by the ethics committee of the National Institute for Biotechnology and Genetic Engineering (NIBGE) in Faisalabad, Pakistan and University of Punjab, Lahore, Pakistan.

### 2.2 Genomic analyses

For disease gene identification, a stepwise approach was followed; first, a few families were directed for Sanger sequencing of *ASPM* due to the fact that it is the most common cause of MCPH in Pakistan as well as worldwide (Ahmad et al., 2017). Second, a few families were sequenced for gene panel customized for screening the reported genes of MCPH (Table S2). Third, families excluded from mutations in *ASPM* were subjected to whole‐exome sequencing (WES) using the Agilent (Santa Clara, CA) version 6 enrichment kit and the Illumina HiSeq 4000 sequencing system (paired‐end reads, 2 x75 bp). WES data were analyzed using our in‐house VARBANK database (http://varbank.ccg.uni‐koeln.de). In addition to the above‐mentioned approaches, a few families were also investigated by genome‐wide linkage analysis using Illumina (San Diego, CA) HumanCoreExome 12 v1.1 array and Affymetrix (Santa Clara, CA) Axiom Precision Medicine Research Array (PMRA), as described earlier (Moawia et al., 2017). WES data analysis provided us with the information of potentially pathogenic variants which were further validated for co‐segregation by Sanger sequencing (Figure S3). Data obtained by linkage analysis by genotyping of only a few selected families corroborated the involvement of the identified gene (Figure S4). The identified variants were further consulted to calculate the allele frequency in multiple databases such as in‐house database of Cologne Center for Genomics with >1600 exomes, dbSNP151, 1000 Genomes (build 20110521), the public Exome Variant Server, NHLBI Exome Sequencing Project (ESP), Seattle (build ESP6500), gnomAD, Iranome, and the Greater Middle Eastern Variome.

## 3 RESULTS

In this study, we report mutational findings of 32 consanguineous Pakistani families with 98 affected individuals (57 males and 41 females)—70 of them were analyzed in this study. The patients were recruited according to the diagnostic criteria of reduced HC (≥3 SD), intellectual disability, and absence of brain atrophy/indicative symptoms. Clinical investigation of all affected individuals revealed reduced skull size (head circumference below −5 SD) and mild to profound intellectual disability. In addition, speech impairment, aggressive behavior, and self‐care deficits were notable features for most of the affected individuals (Figures S1 and S2, and Table S3).

**Figure 1:**
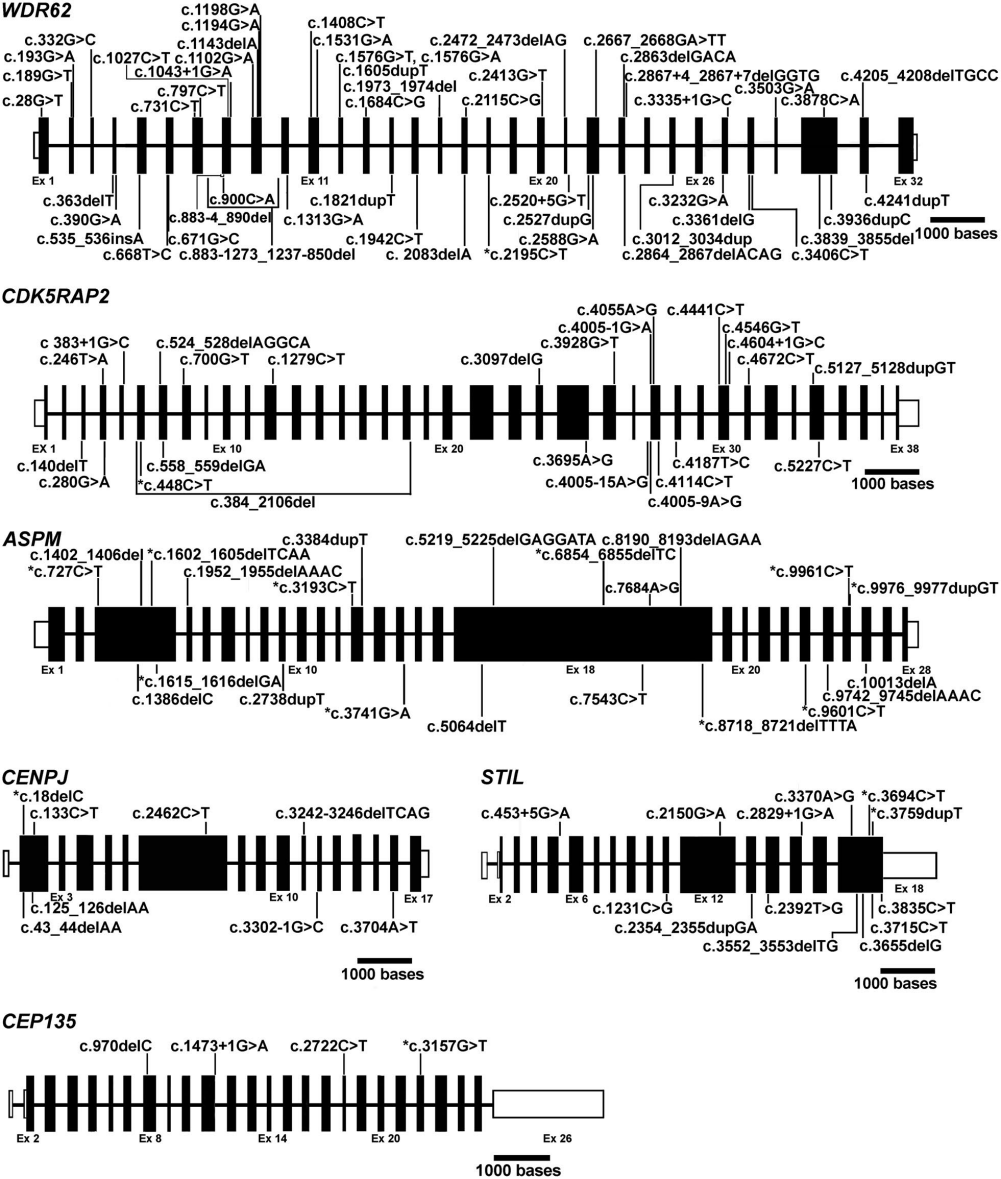
Schematic representation of the genomic structure of human *WDR62*,* CDK5RAP2*,* ASPM*,* CENPJ*, and *STIL* along with the causal variants. Figure shows all the known and novel mutations of *WDR62*,* CDK5RAP2*,* ASPM*,* CENPJ*,* STIL*, and *CEP135* causing primary and syndromic microcephaly due to the mutations in the respective genes. Exons are drawn according to the given scale whereas introns are shown as arbitrary lines. White boxes denote untranslated regions. Notice that asterisk (*) represents the novel mutations found in this study. *ASPM* variant;c.3384dupT, previously known as c.3384_3385insT, is renamed according to the HGVS (Human Genome Variation Society) guidelines

Genetic investigations conducted on 32 families divulged that 22 families had mutations in *ASPM*, 5 in *WDR62*, 1 in *CDK5RAP2* (MIM#608201), 1 in *CENPJ* (MIM#609279), 2 in *STIL* (MIM#181590), and 1 in *CEP135* (MIM#611423) (Figure 1 and Table 1). This information supported the previous observation of *ASPM* and *WDR62* being the most commonly involved genes in MCPH (Ahmad et al., 2017). Notably, our genetic investigations revealed 15 novel mutations in five different MCPH genes; *ASPM* (10), *WDR62* (1), *CDK5RAP2* (1), *STIL* (2), and *CEP135* (1) (Figure 1 and Table 1).

**Table 1:** List of MCPH mutations identified in this study

Gene	Exon	Ref. Seq. ID	cDNA mutation	Protein mutation	gnomAD Frequency^c^	Family ID	Reference
*ASPM*	3	NM_018136.4	c.727C>T	p.(Arg243*)	3.98e−6/1,245848,0	Family 20	Novel
	3	NM_018136.4	c.1260_1266delTCAAGTC	p.(Gln421Hisfs*32)	4e−6/1,245792,0	Family 7,13	Gul et al. (2006)
	3	NM_018136.4	c.1602_1605delTCAA	p.(Asn534Lysfs*14)	0	Family 14	Novel
	3	NM_018136.4	c.1615_1616delGA	p.(Glu539Argfs*15)	0	Family 25	Novel
	13	NM_018136.4	c.3193C>T	p.(Gln1065*)	0	Family 27	Novel
	18	NM_018136.4	c.4212G>A	p.(Trp1404*)	0	Family 18	Ahmad et al. (2017)
	18	NM_018136.4	c.5149delA	p.(Ile1717*)	0	Family 12,19	Gul et al. (2007)
	18	NM_018136.4	c.5959C>T	p.(Gln1987*)	0	Family 30	Ahmad et al. (2017)
	18	NM_018136.4	c.7782_7783delGA	p.(Lys2595Serfs*6)	2.23e−4/62,278516,0	Family 31	Passemard et al. (2016)
	18	NM_018136.4	c.8190_8193delAGAA	p.(Arg2732Lysfs*4)	8.09e−6/2,242788,0	Family 6	Passemard et al. (2009)
	18	NM_018136.4	c.8508_8509delGA	p.(Lys2837Metfs*34)	8.02e−6/2,244284,0	Family 1,5	Bond et al. (2003)
	18	NM_018136.4	c.8718_8721delTTTA	p.(Leu2907Argfs*30)	0	Family 26	Novel
	21	NM_018136.4	c.9190C>T	p.(Arg3064*)	2.8e−5/7,244828,0	Family 15	Nicholas et al. (2009)
	21	NM_018136.4	c.9286C>T	p.(Arg3096*)	4.089e−6/1,249754,0	Family 28	Darvish et al. (2010)
	23	NM_018136.4	c.9557C>G	p.(Ser3186*)	3.99e−6/1,245312,0	Family 17	Gul et al. (2006)
	23	NM_018136.4	c.9601C>T	p.(Gln3201*)	0	Family 22	Novel
	24	NM_018136.4	c.9789 T > A	p.(Tyr3263*)	0	Family 2	Nicholas et al. (2009)
	25	NM_018136.4	c.9961C>T	p.(Gln3321*)	3.99e−6/1,245316,0	Family 16	Novel
	18	NM_018136.4	c.6854_6855delTC	p.(Leu2285Glnfs*32)	2.13e−5/6,281042,0	Family 32	Novel
	25		c.9976_9977dupGT	p.(Ser3327 Tyrfs*14)	NA		Novel
	15		c.3741G>A	p.(Lys1247=)	NA		Novel
*WDR62*	9	NM_001083961.1	c.1194G>A	p.(Trp398*)	1.22e−5/3,245910,0	Family 21	Sajid Hussain et al. (2013)
	18	NM_001083961.1	c.2195C>T	p.(Thr732Ile)	0	Family 4	Novel
	28	NM_001083961.1	c.3361delG	p.(Ala1121Glnfs*6)	0	Family 10	Sajid Hussain et al. (2013)
	30	NM_001083961.1	c.3936dupC^a^	p.(Val1313Argfs*18)	0	Family 8	Yu et al. (2010)
	31	NM_001083961.1	c.4241dupT^b^	p.(Ser1415Glufs*40)	0	Family 9	Nicholas et al. (2010)
*CDK5RAP2*	6	NM_018249.5	c.448C>T	p.(Arg150*)	1.59e−5/4,246038,0	Family 3	Novel
*CENPJ*	2	NM_018451.4	c.18delC	p.(Ser7Profs*2)	0	Family 11	Bond et al. (2005)
*STIL*	17	NM_001048166.1	c.3694C>T	p.(Arg1232*)	1.99e−5/5,246162,0	Family 24	Novel
	17	NM_001048166.1	c.3759dupT	p.(Pro1254Serfs*2)	0	Family 29	Novel
*CEP135*	23	NM_025009.4	c.3157G>T	p.(Glu1053*)	3.99e−6/1,245562,0	Family 23	Novel

^a^ This mutation was previously reported as c.3936_3937insC.

^b^ c.4241_4242insT, both of them were corrected according to the HGVS (Human Genome Variation Society) guidelines.

^c^ Allele frequency observed in gnomAD/allele count, total allele number, number of homozygotes.

Out of 22 *ASPM* linked families, 8 carried 10 novel mutations—seven homozygous in seven families and three heterozygous in one family. All of the seven homozygous mutations were protein‐truncating—four nonsense and three frameshifts—which may result in the complete loss‐of‐function of ASPM. Interestingly, a family with three heterozygous mutations carried two frameshifts (p.(Leu2285Glnfs*32); p.(Ser3327 Tyrfs*14)) and one apparently synonymous substitution (p.(Lys1247=)). We speculate that this synonymous substitution may also disrupt splicing as the altered nucleotide (c.3741G>A) is present at the 5′ end of exon 15 of *ASPM*. The effect of this variation on splicing, however, could not be verified due to the unavailability of RNA from the patient. The remaining 14 families segregated already reported 11 mutations (Table 1) (Ahmad et al., 2017; Bond et al., 2003; Darvish et al., 2010; Gul et al., 2006, 2007; Nicholas et al., 2009; Passemard et al., 2009, 2016). Three of these known mutations were found in two families each (Table 1). The homozygous frameshift mutation c.8190_8193AGAA;p.(Arg2732Lysfs*4) has been previously reported in the compound heterozygous state together with c.3945_3946delATCTT;p.Arg1315Serfs*2 (Passemard et al., 2009). All the patients carrying *ASPM* mutation(s) manifested one or more comorbidities such as seizures, stuttering, hypersalivation, and cup‐shaped ears. One notable additional feature was bilateral hearing impairment (HI), observed in two (out of three) patients of family 14 who carry a protein‐truncating *ASPM* mutation (Table S3). Sensorineural hearing loss has previously been reported in MCPH patients carrying mutations in *ASPM* and *CDK5RAP2* (Darvish et al., 2010; Pagnamenta et al., 2012). To check for the possibility of an independent mutation causing HI in these patients, we interviewed the extended family for a history of HI segregating independently of MCPH, but it was found unremarkable. Parallel to the sequencing approach, genome‐wide linkage analysis was also performed in five selected families on the Axiom Precision Medicine Research Array (PMRA) from Affymetrix (Santa Clara, CA), which delineated the homozygous segments on chromosome 1 harboring *ASPM* (Figure S4), thus providing further evidence for the involvement of this gene in disease causation.

The second most frequently mutated gene (15.62%) in our cohort was *WDR62*. In this gene, five mutations were identified which included one novel missense and four known protein‐truncating variants (Table 1 and Figure 1) (Nicholas et al., 2010; Sajid Hussain et al., 2013; Yu et al., 2010). The missense mutation c.2195C>T;p.(Thr732Ile), identified in family 4, is predicted to be deleterious and disease causing by a number of bioinformatics tools (CADD score = 30, PROVEAN = −4.262, Mutation Taster = 89, PolyPhen‐2 = 0.99 and SIFT = 0.01). Further, this mutation is not documented in any of the public databases of genomic variants. Genome‐wide linkage analysis for selected members of this family revealed a homozygous segment on chromosome 19 at cytoband 19q13.1‐13.3 with a LOD score of 5.4. This homozygous segment (rs73039760‐32,418,599 bp to rs4141695‐44,985,674 bp) contains *WDR62*, the only gene in which a deleterious mutation was identified (Figure S4). In addition to core symptoms of microcephaly (mean HC = −10 SD), mild to moderate intellectual disability, the patients also presented speech impairment, short stature (in V‐6), and seizures (in IV‐1) (Table S3).

Family 3 from our cohort was identified with a novel mutation in *CDK5RAP2*. Affected individuals of this family presented severe microcephaly with HC ranging −11 to −12 SD, speech impairment, and short stature ranging from −5 SD to −6 SD (Table S3). Linkage analysis of this family indicated four peaks reaching the theoretical maximum LOD score of 3.01 on chromosome 8, 9, 13, and 20. The candidate variant obtained from exome sequencing was located in the linkage region on chromosome 9 (Figure S4). It was a homozygous truncating mutation in exon 6 of *CDK5RAP2* (NM_018249.5;c.448C>T;(p.Arg150*)) (Table 1).

One family of our cohort carried a previously reported homozygous frameshift mutation c.18delC;p.(Ser7Profs*2) of *CENPJ* (Bond et al., 2005). We also identified two novel mutations in *STIL*; one nonsense mutation c.3694C>T;p.(Arg1232*) in family 24 and a frameshift mutation c.3759dupT;p.(Pro1254Serfs*2) in family 29 (Figure 1 and Table 1). One family was identified with the nonsense mutation c.3157G>T;p.(Glu1053*) in *CEP135* (Table 1).

## 4 DISCUSSION

*ASPM* encodes abnormal spindle‐like microcephaly‐associated protein and is the most common cause of MCPH contributing 40% to 68% of MCPH cases (Letard et al., 2018; Zaqout et al., 2017). The findings of this study are in line with previous reports because we have also observed 68.75% (22/32) families carrying a mutation in *ASPM*. Building on another elegant study by Letard and colleagues (Letard et al., 2018)—summarizing 189 *ASPM* mutations in 282 previously reported and 39 new families—we reviewed the literature published thereafter and extended the mutational spectrum to 211 in 381 families (Ahmed et al., 2019; Bazgir, Agha Gholizadeh, Sarvar, & Pakzad, 2019; Bhargav, Sreedevi, Swapna, Vivek, & Kovvali, 2017; Boonsawat et al., 2019; Khan, Wang, Han, Ahmad, & Zhang, 2018; Kvarnung et al., 2018; Li et al., 2017; Marakhonov et al., 2018; McSherry et al., 2018; Moriwaki et al., 2019; Okamoto, Kohmoto, Naruto, Masuda, & Imoto, 2018; Shaheen et al., 2019). This number also includes 10 novel mutations of 8 families identified in this study (Figure 1 and Table S4).

Intriguingly, our cohort did not show the previously identified founder mutation, c.3978G; p.(Trp1326*), of Northern Pakistani Pashtun ethnicity (Ahmad et al., 2017). One explanation for this discrepancy is that none of the families recruited for this study belonged to Pashtun ethnicity; rather they originated from a region with population composition of diverse ethnic background. Thus our findings support this mutation to be a founder event, rather than a recurrent mutation. It is, however, noteworthy that our cohort contains a family carrying another recurrent mutation—c.7782_7783delGA;p.(Lys2595Serfs*6)—of *ASPM*, which has been reported in several different ethnic groups originating from Europe, Africa, and Asia (Letard et al., 2018). Another nonsense mutation, *ASPM*:c.9557C>G;p.(Ser3186*), reported exclusively in even Pakistani families, is also found in a family from our cohort (Table 1 and Figure 1), which increases the number of families with this particular mutation from 7 to 8. This observation indicated that it could be another founder mutation in the Pakistani population.

Our data also supported the notion that *WDR62* (MIM# 613583) is the second most frequently mutated gene (14%) of MCPH (Zaqout et al., 2017), because we have found a nearly similar number of MCPH families (15.62%) with mutations in this gene. After a thorough literature review of *WDR62* mutations by Poulton and colleagues (Poulton et al., 2014), summarizing a total of 24 mutations, 29 new mutations were published thereafter (Kvarnung et al., 2018; Yi et al., 2019; Zombor et al., 2019). The novel missense mutation reported in our study increased the total number of identified mutations to 54 (Figure 1 and Table S5).

So far, 24 mutations of 24 families have been reported in *CDK5RAP2* and the identification of the novel nonsense mutation in this study increased this number to 25 (Figure 1 and Table S6) (Ahmad et al., 2017; Issa et al., 2013; Shaheen et al., 2019). A few cases of *ASPM* and *CDK5RAP2*‐related MCPH have been reported with short stature at birth, but the majority of them attained normal height at a later age (Issa et al., 2013; Passemard et al., 2009). Our patients show short stature at the age of 10 years, which adds to the phenotypic spectrum due to *CDK5RAP2* dysfunction. Notably, one of the patients (V‐6) of family 4 mutated with *WDR62* also show short stature clinically diagnosed only at the age of 18 years. This is another example of a patient unable to attain normal height at adult age. The literature survey revealed only eight mutations of *CENPJ* found in 17 families, including one reported in this study, manifesting the MCPH phenotype (Figure 1 and Table S7) (Darvish et al., 2010; Sajid Hussain et al., 2013; Shaheen et al., 2019). This frameshift mutation could reasonably be a founder mutation of the Pakistani population as this is the 5^th^ consecutive Pakistani family reported with this mutation. Analysis of published data about disease‐causing *STIL* mutations and by including two novel mutations found in this study raise the total number of *STIL* mutations to 13 in 12 families (Figure 1 and Table S8) (Cristofoli, De Keersmaecker, De Catte, Vermeesch, & Van Esch, 2017; Shaheen et al., 2019). Nonsense mutation of *CEP135* could most likely cause nonsense‐mediated decay of the mutant transcript; else it would result in a truncated protein, lacking the C‐terminus. Hitherto, only three mutations have been reported in *CEP135* and our report of this additional one raises this number to 4 in four families (Figure 1 and Table S9) (Farooq et al., 2016; Hussain et al., 2012; Shaheen et al., 2019).

Conclusively, in 32 families from Pakistan, we have identified 15 novel variants and 16 previously reported ones in well‐characterized MCPH genes (Table 1). Functional evaluation of the novel variants will elucidate their effect on neurogenesis and the development of the MCPH phenotype. It will also help to develop correlation with the type of mutation and the severity of the disease. Finally, the review of published variants and addition of pathogenic variants of MCPH genes provided by this study will be helpful for devising diagnostic strategies for MCPH.

## CONFLICT OF INTEREST

No competing interests.

## AUTHOR CONTRIBUTIONS

Sajida Rasool, Jamshaid Mahmood Baig, Abubakar Moawia: Methodology, investigation, and validation. Ilyas Ahmad, Maria Iqbal, Syeda Seema Waseem, Maria Asif, Emrah Kaygusuz: Genomic investigation. Uzma Abdullah: Data acquisition, manuscript review, and editing. Ehtisham Ul Haq Makhdoom, Muhammad Zakaria, Shafaq Ramzan, Saif ul Haque, Asif Mir, Iram Anjum, Mehak Fiaz, Zafar Ali, Muhammad Tariq, Neelam Saba, Wajid Hussain, Saba Irshad: Clinical data acquisition. Birgit Budde: Genomic investigations and validation. Angelika Anna Noegel, Stefan Höning, Shahid Mahmood Baig, Peter Nürnberg: Supervision, funding acquisition, and manuscript review and editing. Muhammad Sajid Hussain: Supervision, conceptualization, project administration, visualization and interpretation of data, and manuscript writing.

## Supporting information

Fig S1‐S4

Table S1‐S9
