# Supplementary material for: An update of pathogenic variants in ASPM, WDR62, CDK5RAP2, STIL, CENPJ, and CEP135 underlying autosomal recessive primary microcephaly in 32 consanguineous families from Pakistan
Source: Mol Genet Genomic Med. 2020 Jul 17;8(9):e1408. doi: 10.1002/mgg3.1408 (PMC7507472; doi:10.1002/mgg3.1408)
Supplement: Supplementary file 1 — Fig S1‐S4 [file MGG3-8-e1408-s001.pdf]

FIGURE S1

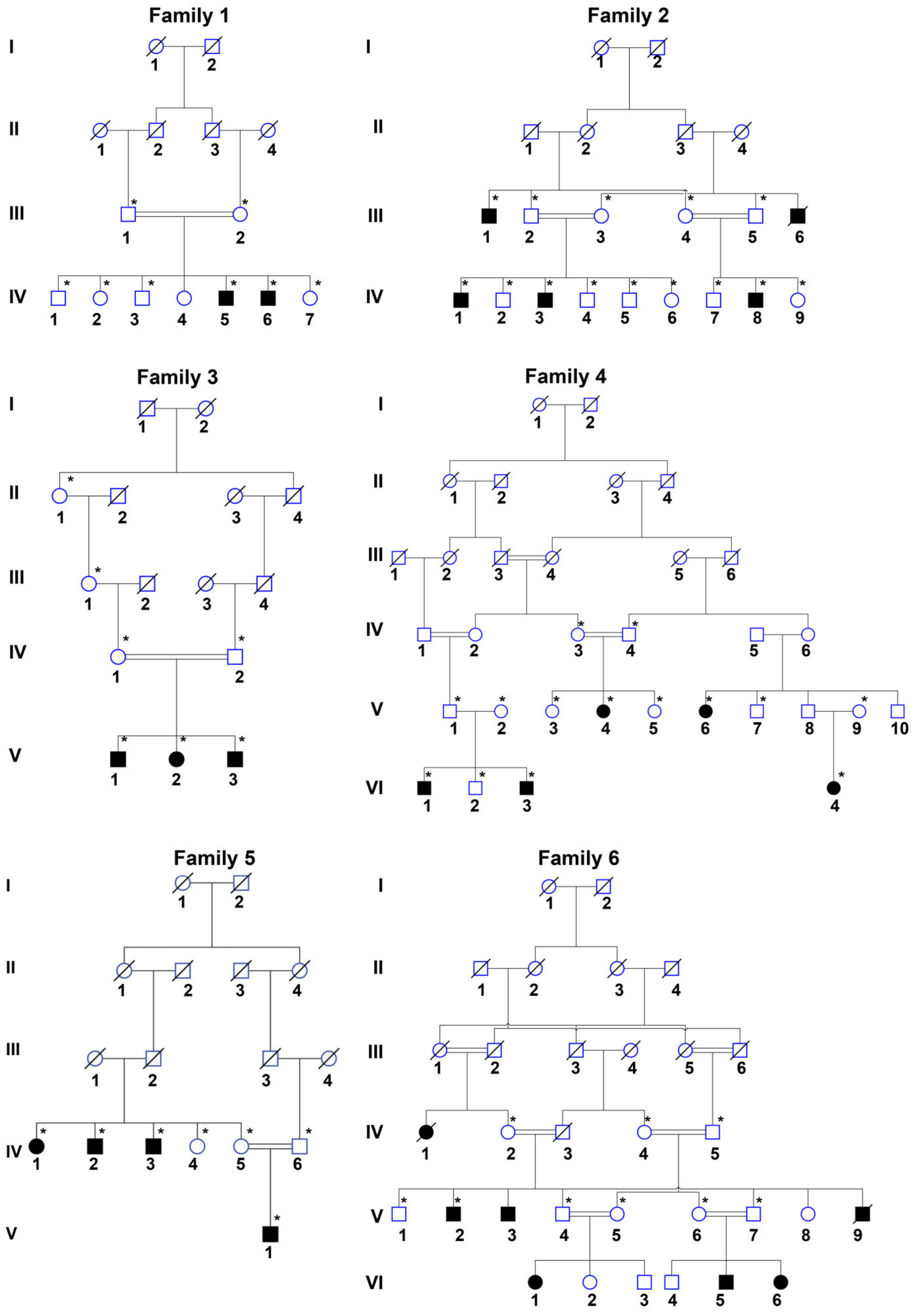

FIGURE S1

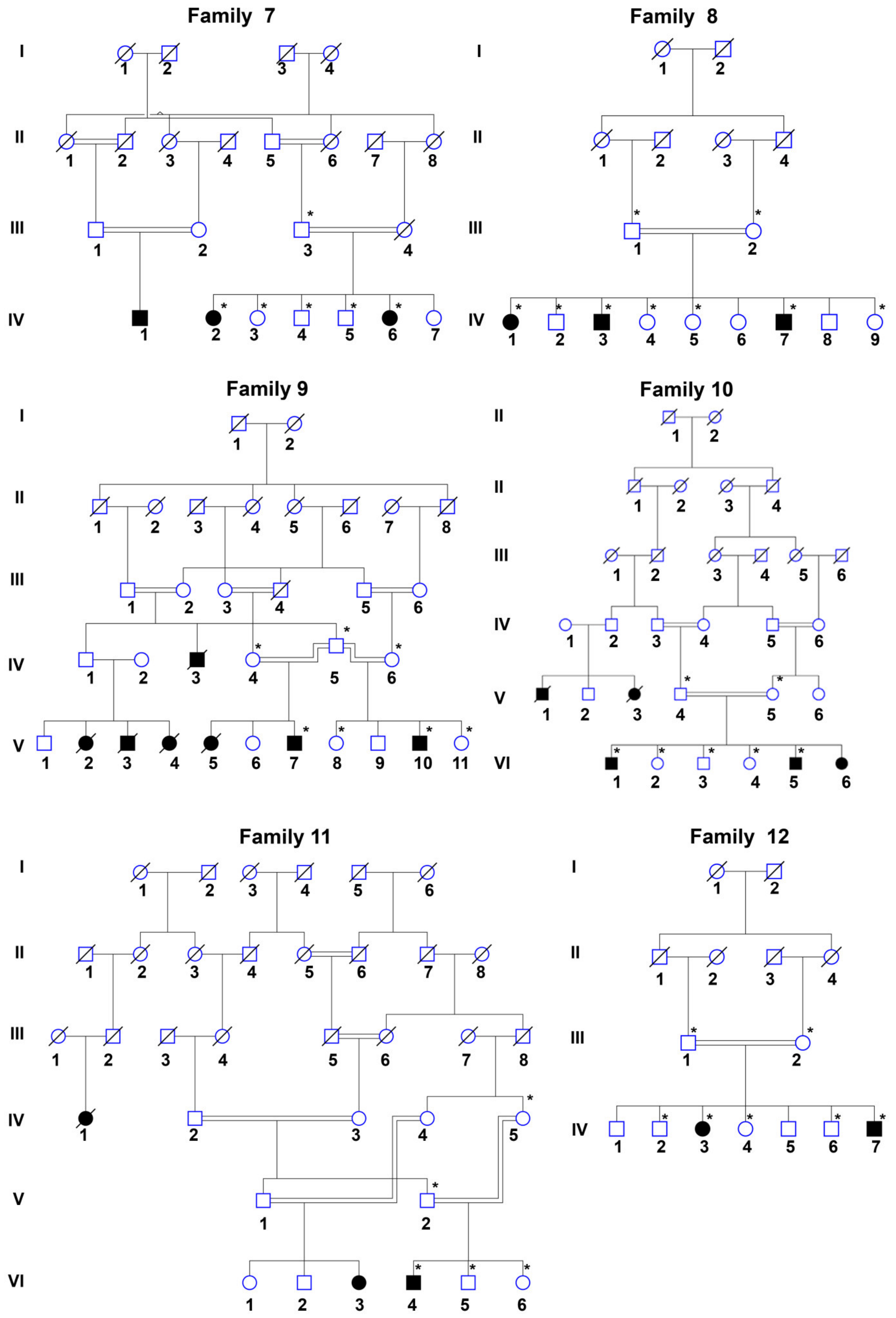

FIGURE S1

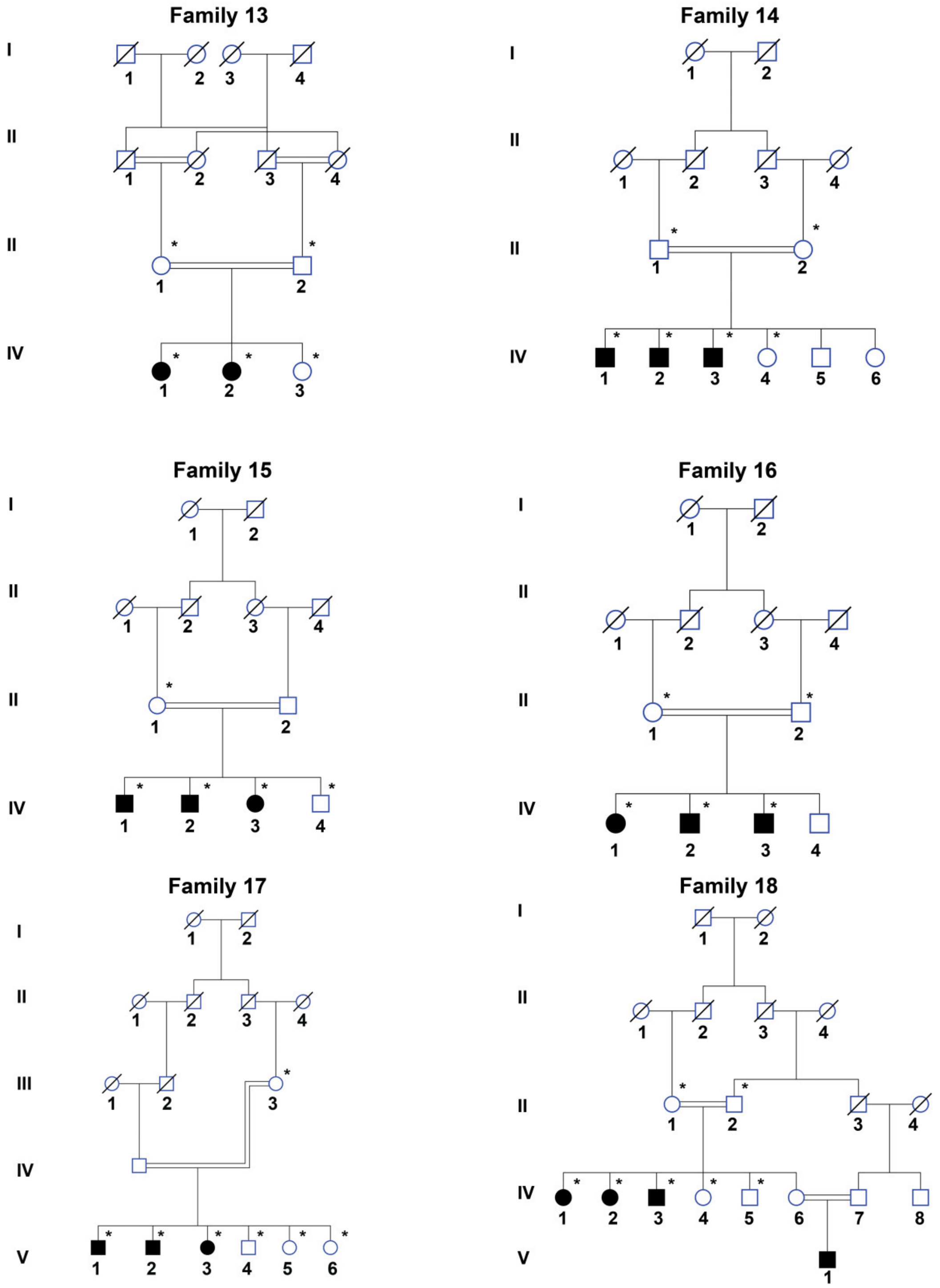

FIGURE S1

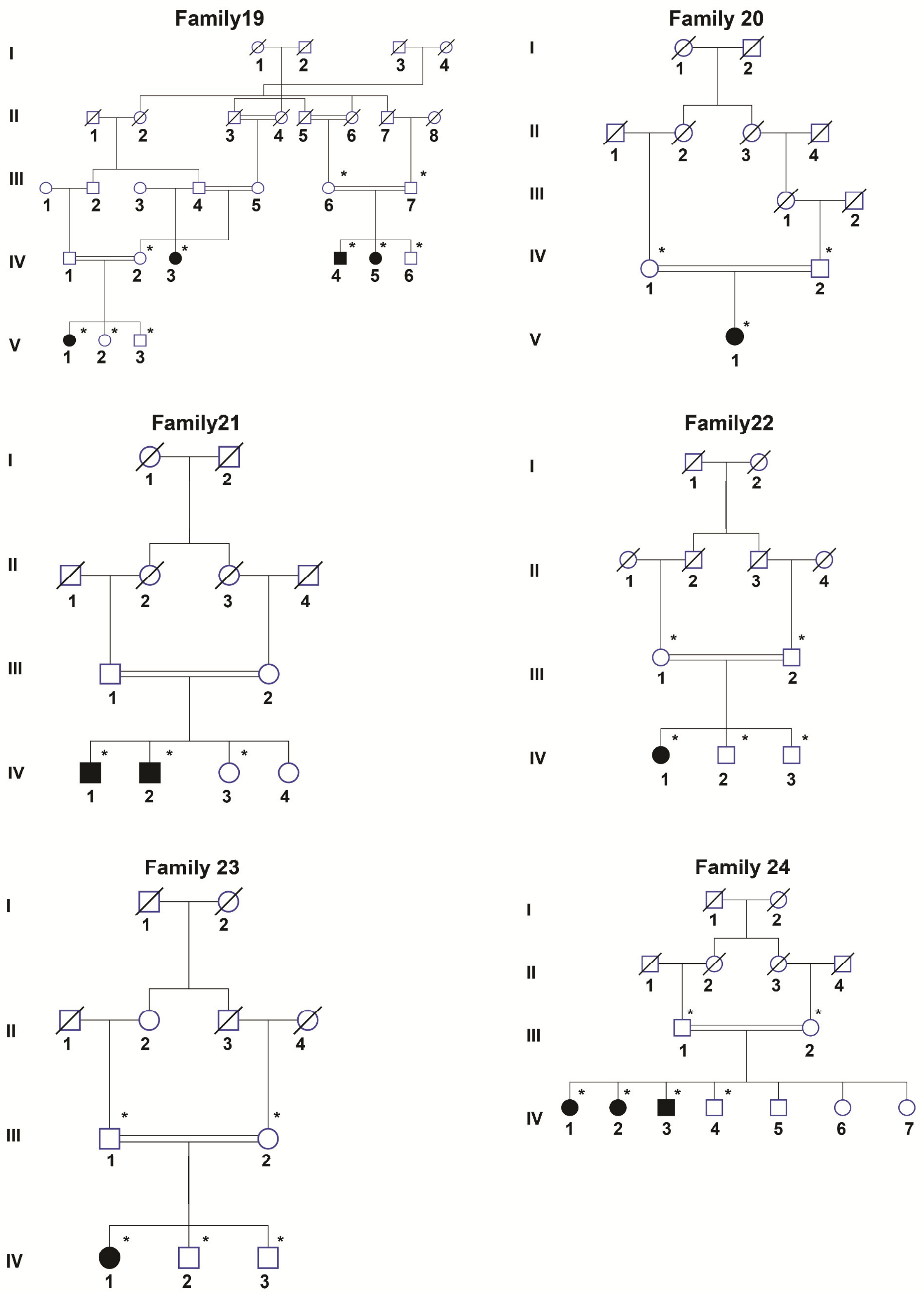

FIGURE S1

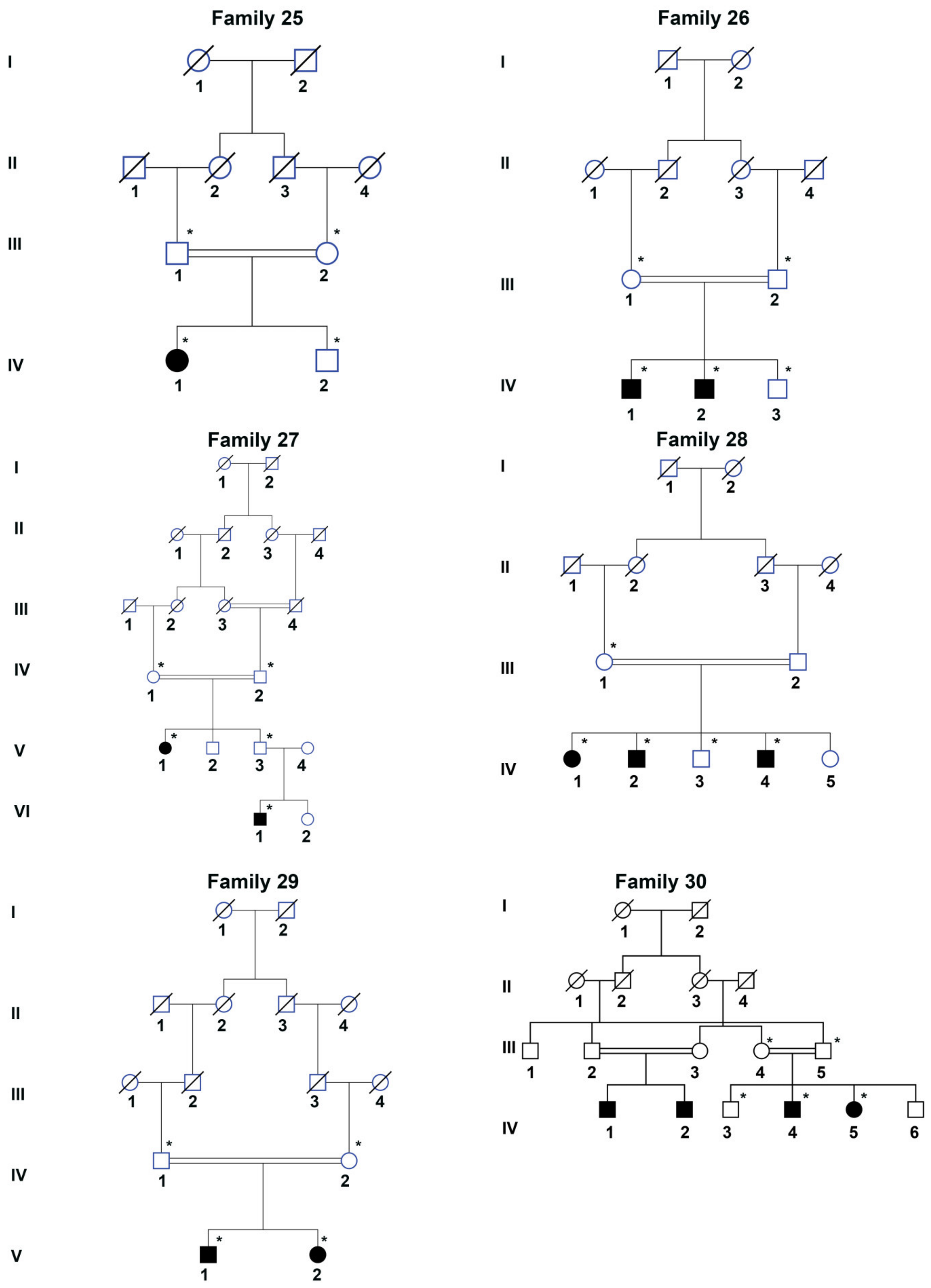
Family 25
Family 26
Family 27
Family 28
Family 29
Family 30
I
II
III
IV
V
VI

FIGURE S1

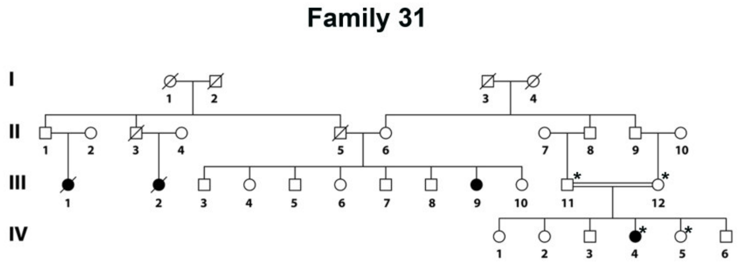

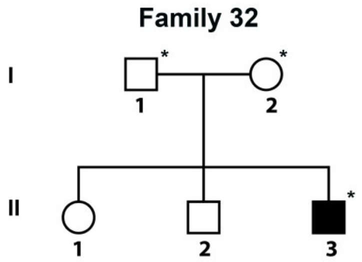

FIGURE S1 Pedigrees of 32 MCPH families subjected for genomic analyses. Asterisks (*) denote individuals participated in this study

FIGURE S2

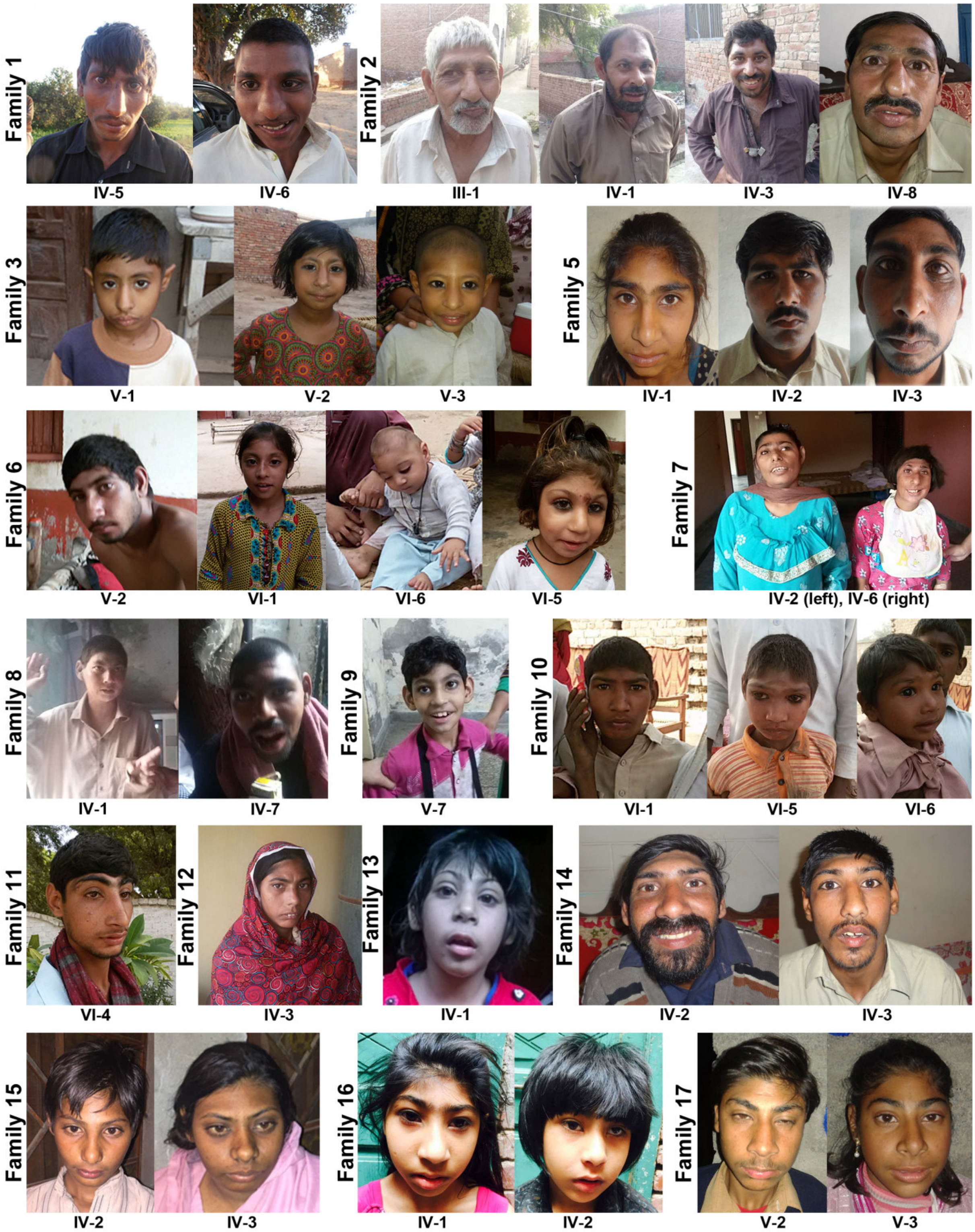
Family 1
IV-5
IV-6
Family 2
III-1
IV-1
IV-3
IV-8
Family 3
V-1
V-2
V-3
Family 5
IV-1
IV-2
IV-3
Family 6
V-2
VI-1
VI-6
VI-5
Family 7
IV-2 (left), IV-6 (right)
Family 8
IV-1
IV-7
Family 9
V-7
Family 10
VI-1
VI-5
VI-6
Family 11
VI-4
Family 12
IV-3
Family 13
IV-1
Family 14
IV-2
IV-3
Family 15
IV-2
IV-3
Family 16
IV-1
IV-2
Family 17
V-2
V-3

FIGURE S2

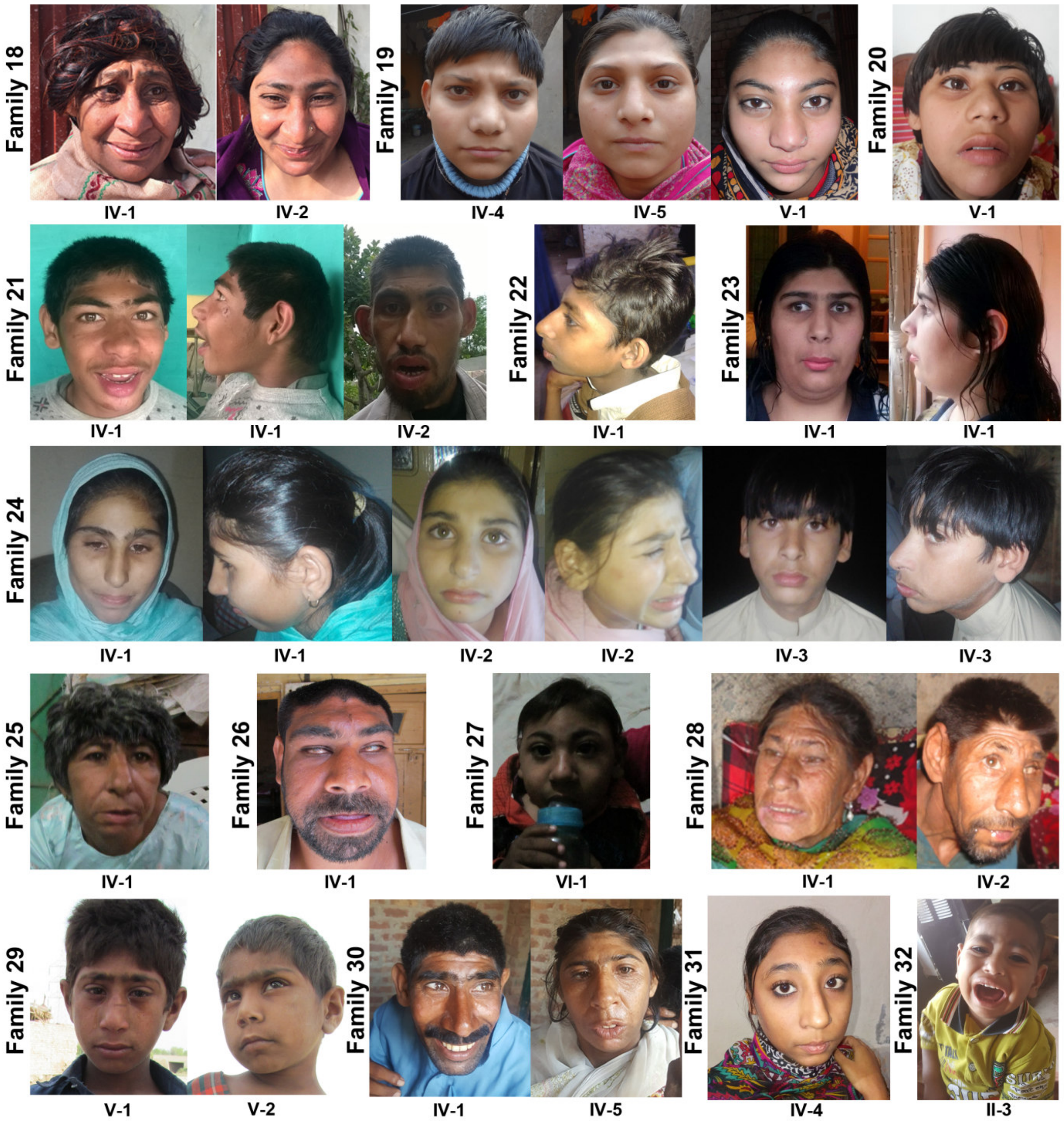

FIGURE S2 Photographs of 32 MCPH Pakistani families manifesting primary microcephaly

FIGURE S3

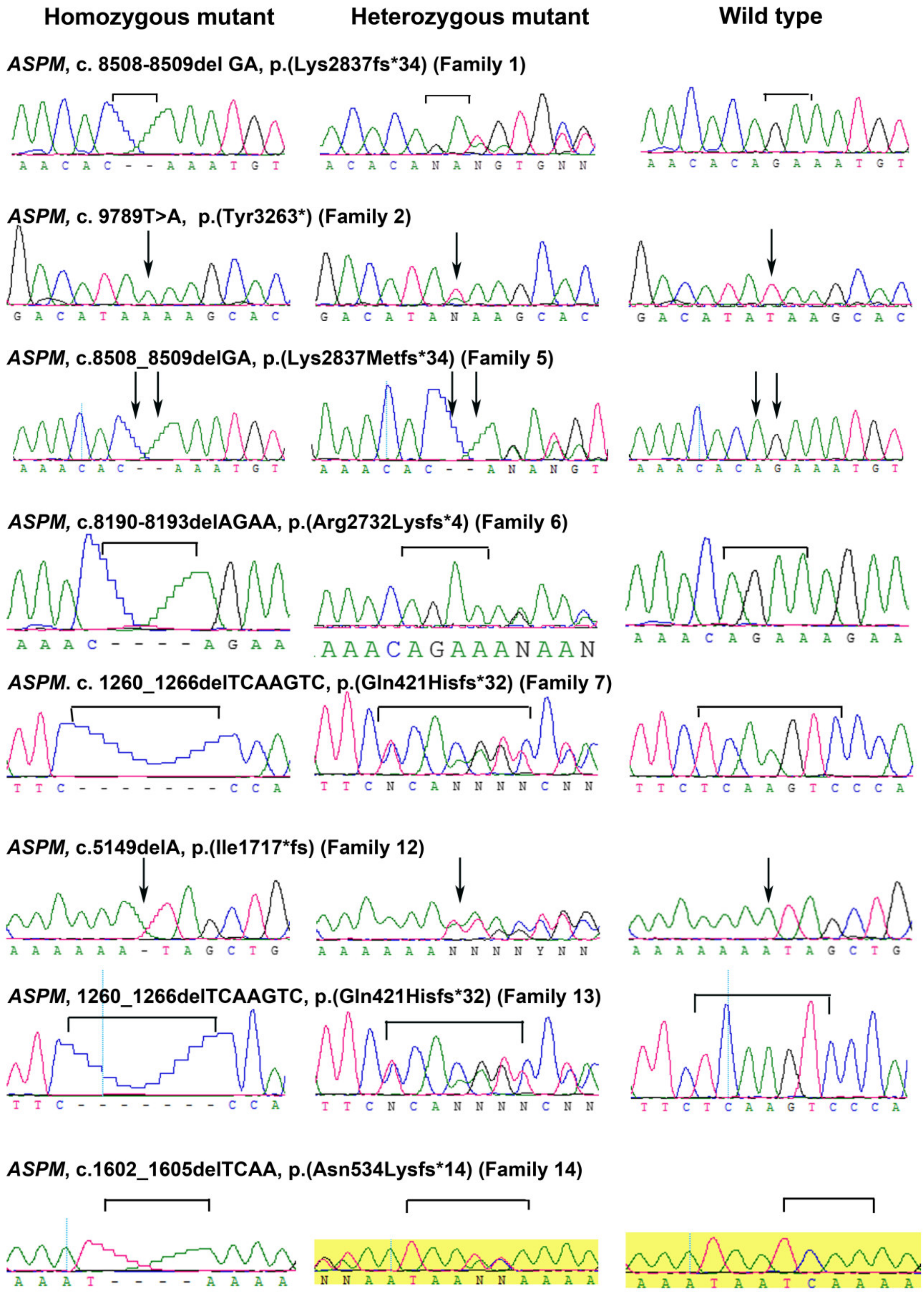
Homozygous mutant
Heterozygous mutant
Wild type
ASPM, c. 8508-8509del GA, p.(Lys2837fs*34) (Family 1)
ASPM, c. 9789T>A, p.(Tyr3263*) (Family 2)
ASPM, c.8508_8509delGA, p.(Lys2837Metfs*34) (Family 5)
ASPM, c.8190-8193delAGAA, p.(Arg2732Lysfs*4) (Family 6)
ASPM. c. 1260_1266delTCAAGTC, p.(Gln421Hisfs*32) (Family 7)
ASPM, c.5149delA, p.(Ile1717*fs) (Family 12)
ASPM, 1260_1266delTCAAGTC, p.(Gln421Hisfs*32) (Family 13)
ASPM, c.1602_1605delTCAA, p.(Asn534Lysfs*14) (Family 14)

FIGURE S3

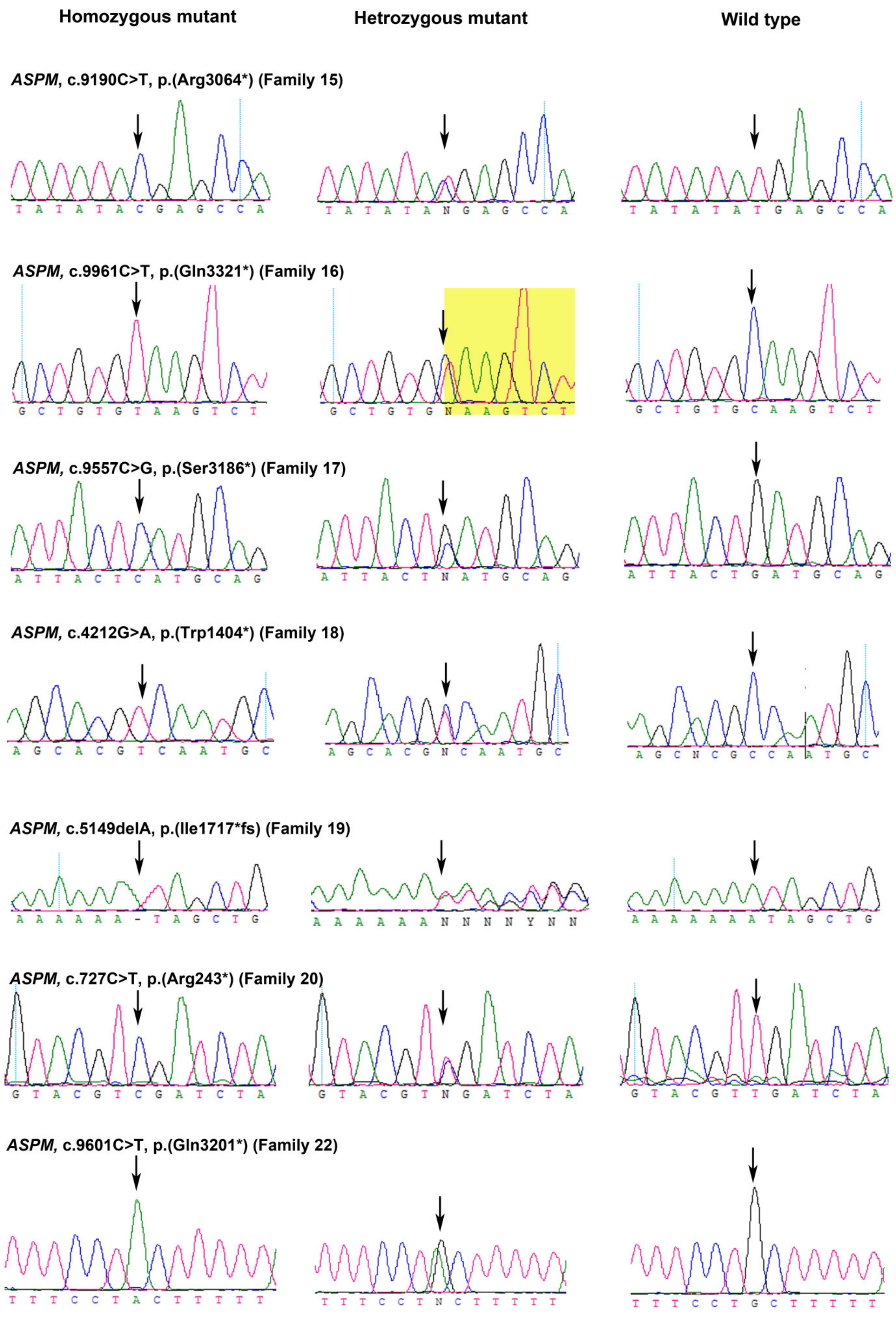
Homozygous mutant
Hetrozygous mutant
Wild type
ASPM, c.9190C>T, p.(Arg3064*) (Family 15)
T A T A T A C G A G C C A
T A T A T A N G A G C C A
T A T A T A T G A G C C A
ASPM, c.9961C>T, p.(Gln3321*) (Family 16)
G C T G T G T A A G T C T
G C T G T G N A A G T C T
G C T G T G C A A G T C T
ASPM, c.9557C>G, p.(Ser3186*) (Family 17)
A T T A C T C A T G C A G
A T T A C T N A T G C A G
A T T A C T G A T G C A G
ASPM, c.4212G>A, p.(Trp1404*) (Family 18)
A G C A C G T C A A T G C
A G C A C G N C A A T G C
A G C N C G C C A A T G C
ASPM, c.5149delA, p.(Ile1717*fs) (Family 19)
A A A A A A - T A G C T G
A A A A A A N N N N Y N N
A A A A A A A T A G C T G
ASPM, c.727C>T, p.(Arg243*) (Family 20)
G T A C G T C G A T C T A
G T A C G T N G A T C T A
G T A C G T T G A T C T A
ASPM, c.9601C>T, p.(Gln3201*) (Family 22)
T T T C C T A C T T T T T
T T T C C T N C T T T T T
T T T C C T G C T T T T T

FIGURE S3

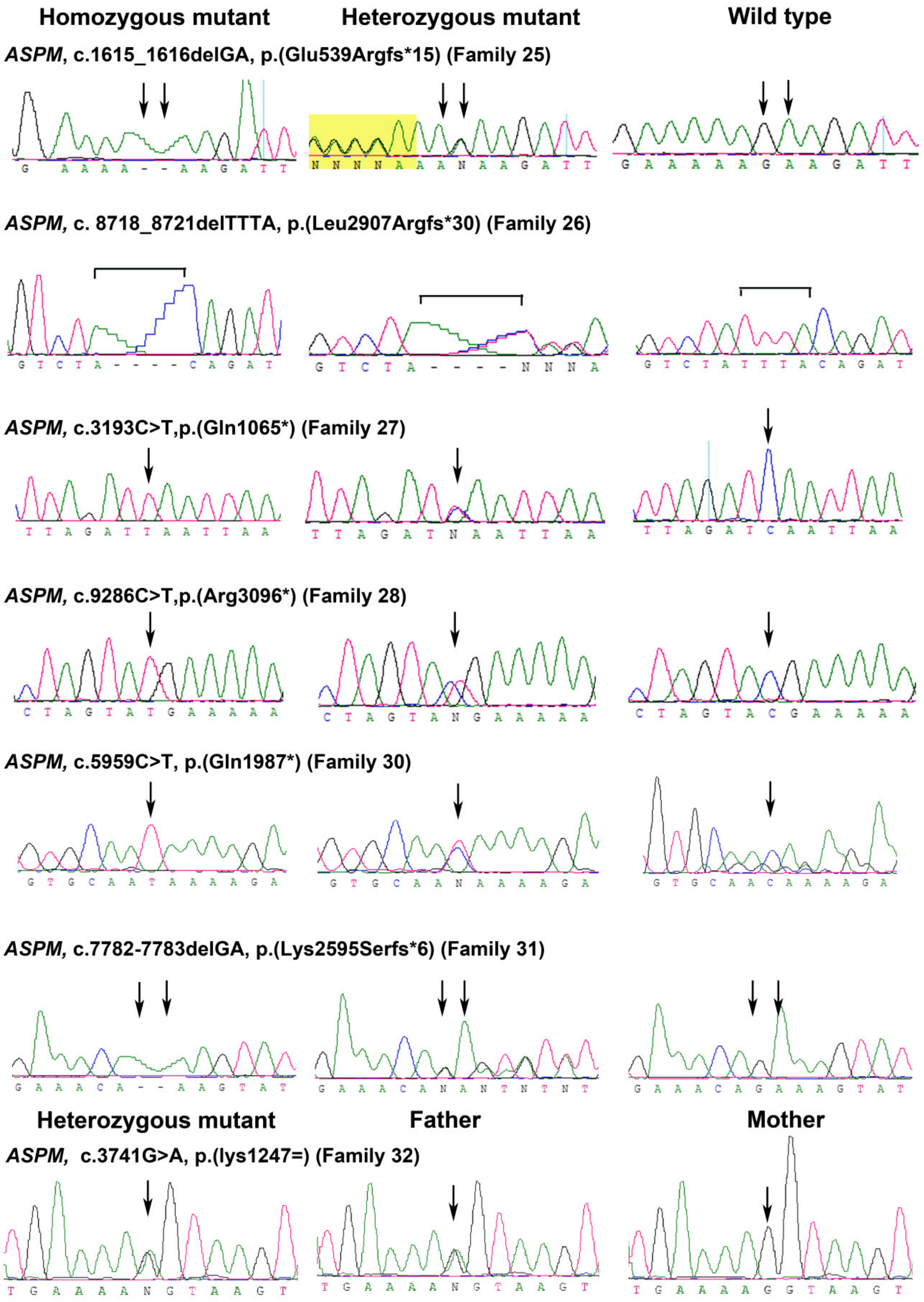
Homozygous mutant
Heterozygous mutant
Wild type
ASPM, c.1615_1616delGA, p.(Glu539Argfs*15) (Family 25)
ASPM, c. 8718_8721delTTTA, p.(Leu2907Argfs*30) (Family 26)
ASPM, c.3193C>T,p.(Gln1065*) (Family 27)
ASPM, c.9286C>T,p.(Arg3096*) (Family 28)
ASPM, c.5959C>T, p.(Gln1987*) (Family 30)
ASPM, c.7782-7783delGA, p.(Lys2595Serfs*6) (Family 31)
Heterozygous mutant
Father
Mother
ASPM, c.3741G>A, p.(lys1247=) (Family 32)

FIGURE S3

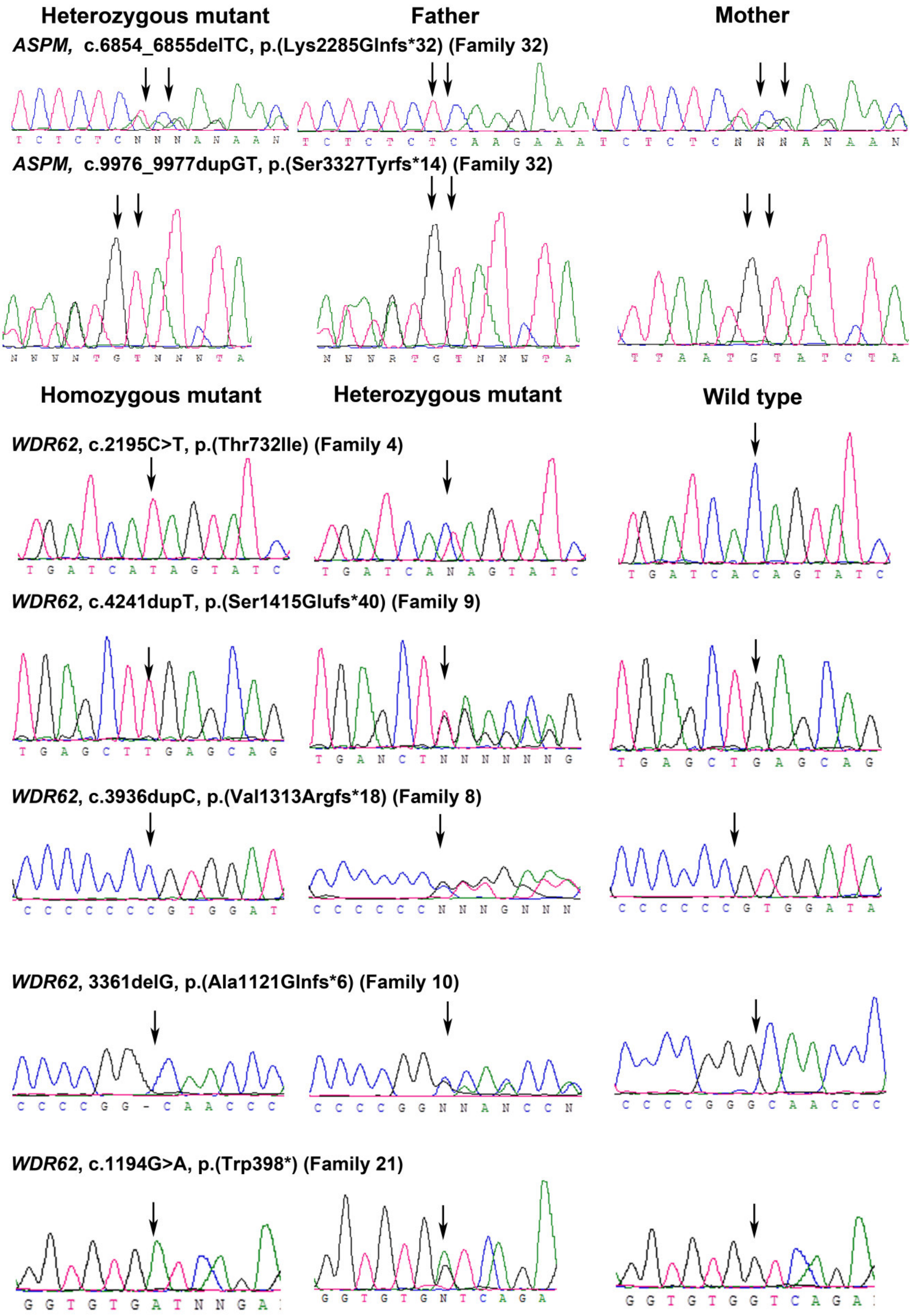
Heterozygous mutant
Father
Mother
ASPM, c.6854_6855delTC, p.(Lys2285Glnfs*32) (Family 32)
ASPM, c.9976_9977dupGT, p.(Ser3327Tyrfs*14) (Family 32)
Homozygous mutant
Heterozygous mutant
Wild type
WDR62, c.2195C>T, p.(Thr732Ile) (Family 4)
WDR62, c.4241dupT, p.(Ser1415Glufs*40) (Family 9)
WDR62, c.3936dupC, p.(Val1313Argfs*18) (Family 8)
WDR62, 3361delG, p.(Ala1121Glnfs*6) (Family 10)
WDR62, c.1194G>A, p.(Trp398*) (Family 21)

FIGURE S3

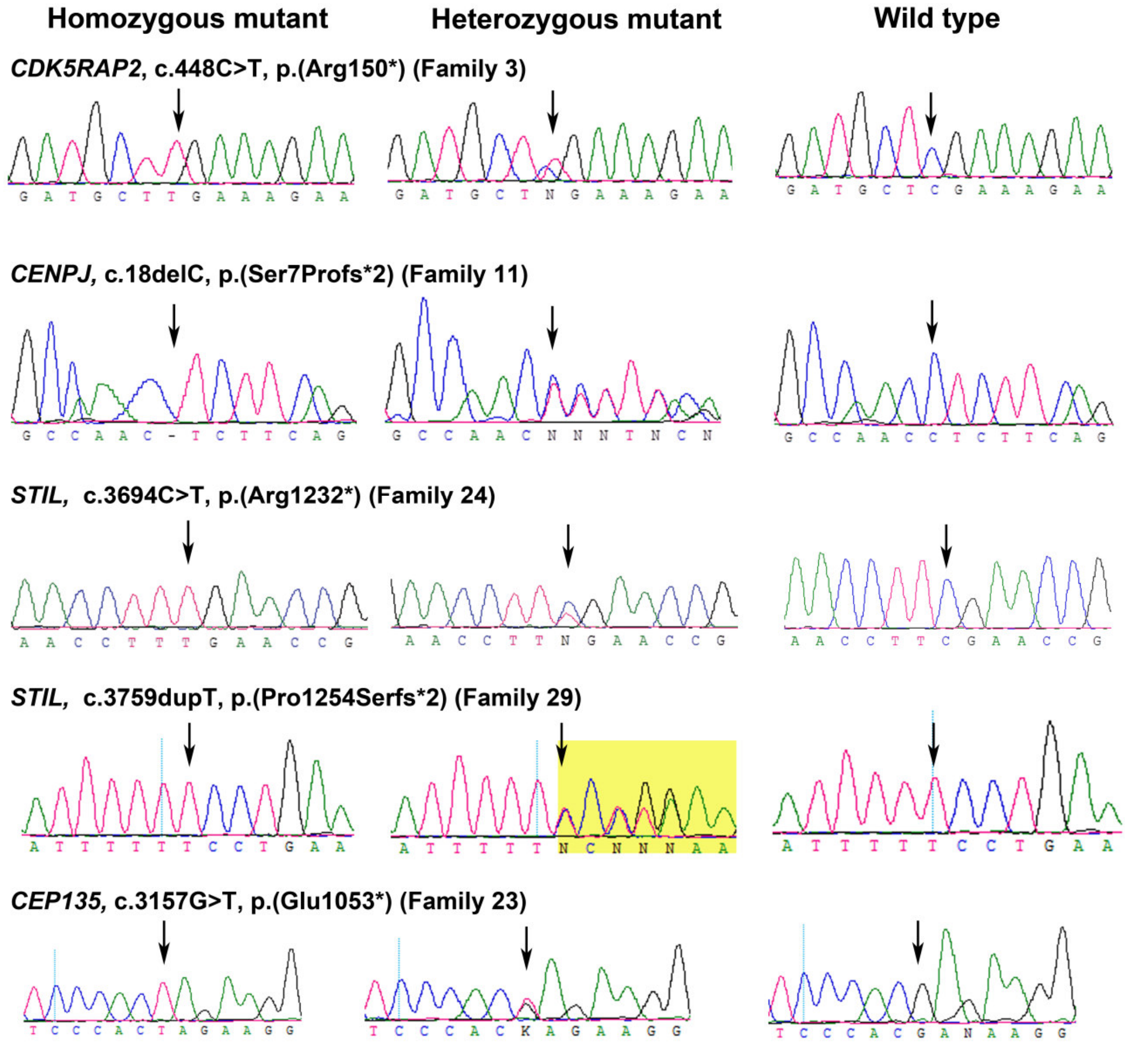

FIGURE S3 Sanger traces of identified mutations in *ASPM, WDR62, CDK5RAP2, CENPJ, STIL* and *CEP135*

FIGURE S4

Linkage region on HSA 9q33-34.1 with LOD score 3.01; rs62574775 to rs4962059 (120,770,009 bp-135,125,789 bp)

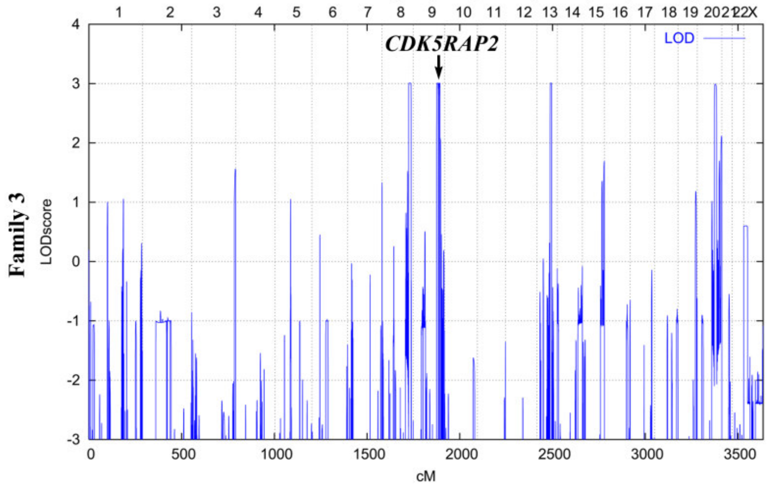

Linkage region on HSA 19q13.1-13.3 with LOD score 5.4; rs73039760 to rs4141695 (32,418,599bp-44,985,674bp)

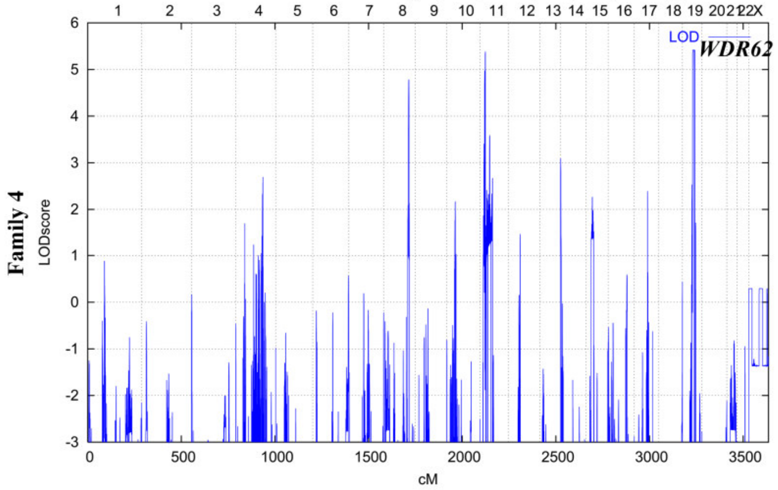

Linkage region on HSA 1q25.3-q32-1 with LOD score 3.0; rs10800790 to rs2500097 (183,765,059 bp-201,644,752 bp)

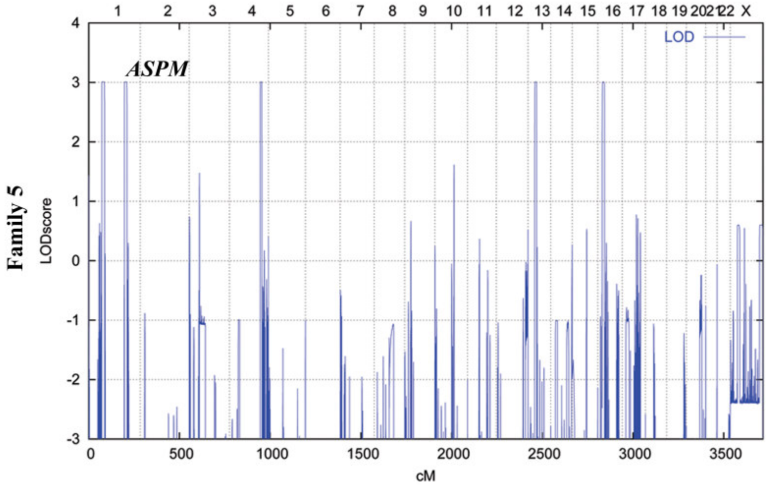

FIGURE S4

Linkage region on HSA 1q31.3-q32.1 with LOD score 2.3;
rs10754124 to rs4951373 (195,024,131 bp-204,423,379 bp)

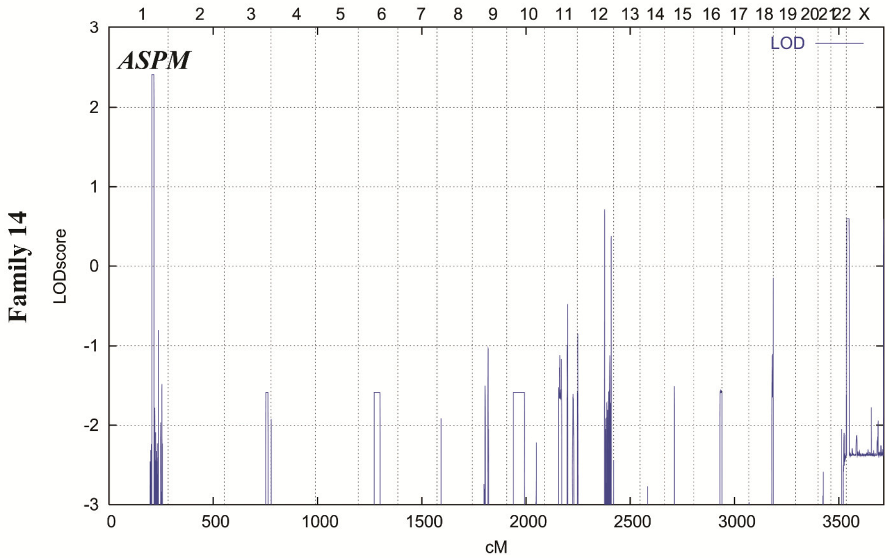

Linkage region on HSA 1q24.3-q32.1 with LOD score 1.9;
rs2223610 to rs4950826 (170,988,745 bp-202,109,049 bp)

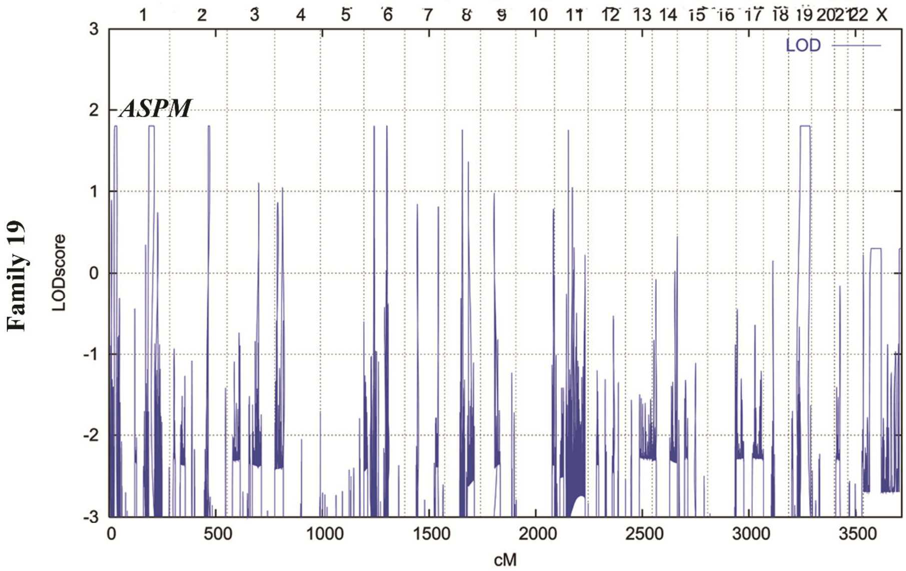

Linkage region on HSA 1q31.3-q32.1 with LOD score 1.33;
rs10900421 to rs9662128 (204,676,917bp-39,684,734 bp)

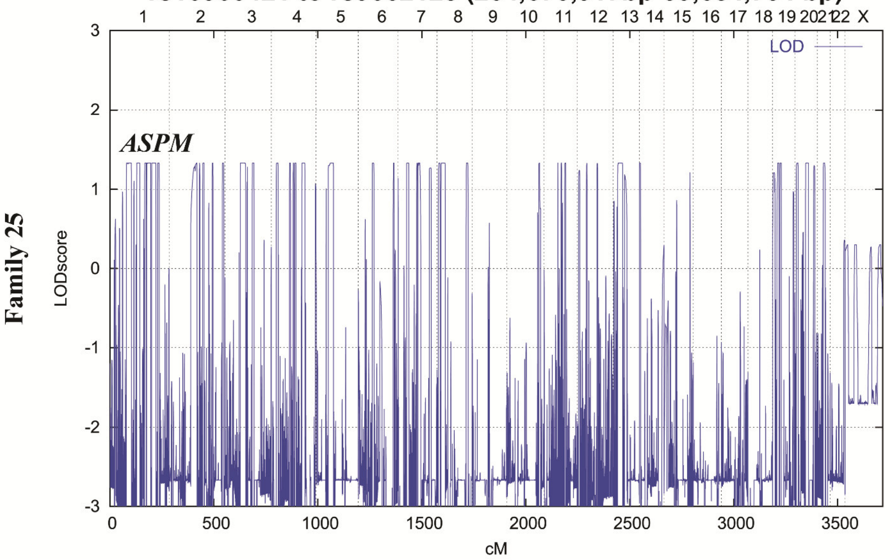

FIGURE S4

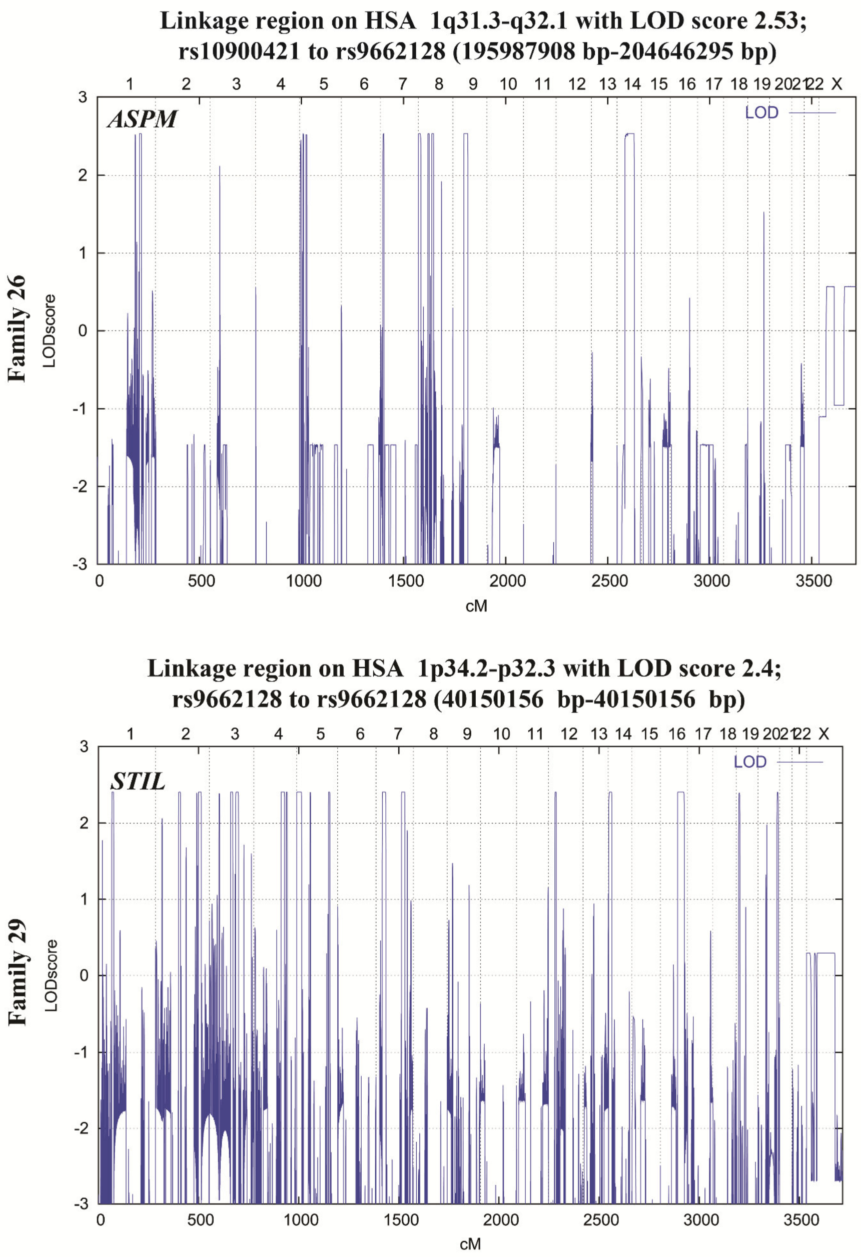

FIGURE S4 Genome-wide graphical views showing the homozygous segments along with the location of the respective causative gene at specific chromosomal region
