# Supplementary material for: An update of pathogenic variants in ASPM, WDR62, CDK5RAP2, STIL, CENPJ, and CEP135 underlying autosomal recessive primary microcephaly in 32 consanguineous families from Pakistan
Source: Mol Genet Genomic Med. 2020 Jul 17;8(9):e1408. doi: 10.1002/mgg3.1408 (PMC7507472; doi:10.1002/mgg3.1408)
Supplement: Supplementary file 2 — Table S1‐S9 [file MGG3-8-e1408-s002.pdf]

TABLE S1 MCPH associated genes and the localization of their respective proteins

**Gene**	**Protein**	**Cellular localization**	**References**
MCPH1, CDK5RAP2, CENPJ, STIL, CEP135, CEP152, CDK6 SASS6 and *CENPE*	MCPH1, CDK5RAP2, CENP-J, STIL, CEP135, CEP152, CDK6, HsSAS-6 and CENP-E	Centrosome	(Bond et al., 2005; Guernsey et al., 2010; Hussain et al., 2012; Hussain et al., 2013; Jackson et al., 2002; Jeffers, Coull, Stack, & Morrison, 2008; M. A. Khan et al., 2017; Kumar, Girimaji, Duvvari, & Blanton, 2009)
WDR62, ASPM, CENPE and *MAP11*	WDR62, ASPM, CENP-E and MAP11	Spindle pole	(Nicholas et al., 2010; Perez et al., 2019; Shen et al., 2005)
KNL1, CENPE and *NUP37*	KNL1, CENP-E and NUP37	Kinetochore	(Braun et al., 2018; Genin et al., 2012; Mirzaa et al., 2014)
ZNF335, PHC1, NCAPD2, NCAPD3, and *NCAPH*	ZNF335, PHC1, Condensin complex subunit 1 (hCAP-D2), Condensin-2 complex subunit D3 (hCAP-D3), Condensin complex subunit 2 (hCAP-H)	Components of Chromatin-remodeling complex/Nucleus	(Awad et al., 2013; Martin et al., 2016; Yang et al., 2012)
MFSD2A, ANKLE2, and *COPB2*	NLS1, Ankyrin repeat and LEM domain-containing protein 2, Coatomer subunit beta (Beta'-COP)	Plasma membrane	(DiStasio et al., 2017; Guemez-Gamboa et al., 2015; Yamamoto et al., 2014)
WDFY3, NCAPD2, and *NCAPH*	WD repeat and FYVE domain-containing protein 3 (Alfy), Condensin complex subunit 1 (hCAP-D2), Condensin complex subunit 2 (hCAP-H)	Cytosol	(Kadir et al., 2016; Martin et al., 2016)
CIT, KIF14 and *MAP11*	CIT, KIF14 and MAP11	Cleavage furrow and midbody of dividing cells	(Basit et al., 2016; Moawia et al., 2017; Perez et al., 2019) 2019)
NUP37	NUP37	Nuclear envelope	(Braun et al., 2018)

TABLE S2 List of genes included in gene panel for next generation sequencing

MCPH1	*TBC1D20*
WDR62	*RAD50*
CDK5RAP2	*CASK*
CASC5	*SLC25A19*
ASPM	*SLC9A6*
CENPJ	*MECP2*
STIL	*TUBGCP6*
CEP135	*ARFGEF2*
CEP152	*TRAPPC9*
ZNF335	*TSEN2*
PHC1	*TSEN34*
CDK6	*TSEN54*
SASS6	*MED17*
NDE1	*CDKL5*
CKAP2L	*ATRX*
ORC1	*FOXG1*
ORC4	*TCF4*
ORC6	*DYRK1A*
CDT1	*KIF11*
CDC6	*SLC2A1*
ATR	*UBE3A*
RBBP8	*ZEB2*
ATRIP	*WDR81*
RNU4ATAC	*CEP290*
CENPE	*OFD1*
DNA2	*CCP110*
PCNT	*CEP97*
CEP63	*CEP76*
NIN	*CNTROB*
PLK4	*POC5*
XRCC4	*SPICE1*
IGF1	*KIAA1279*
LIG4	*HMGB3*
CRIPT	*PQBP1*
IER3IP1	*UBE3B*
TRMT10A	*FAR1*
STAMBP	*NSDHL*
NBN	*ZBTB18*
NHEJ1	*CEP120*
PNKP	*CEP192*
RAB18	*NLRP2*
RAB3GAP1	*QARS*
RAB3GAP2	*WDR73*

TABLE S3 Clinical manifestations of the studied MCPH cohort

Family - Individual	Gene	Mutation information	No. of affected individuals	Age at examination (years)	OFC (cm)	OFC (SD)	Observed clinical manifestation
**Fam.1**	*ASPM*	NM_018136.4;c.8508_8509delGA;p.(Lys2837Metfs*34)	2				
-IV-5				16	43	-8	-Mild level of mental retardation, normal motor skills, normal speech and health
-IV-6				14	45.5	-6	-Mild level of intellectual disability, speech impairment, normal motor skills
**Fam. 2**	*ASPM*	NM_018136.4;c.9789T>A;p.(Tyr3263*)	5				
-III-1				55	43	-8.2	-Mild mental retardation, can speak few words, normal motor skills, no seizures, self-care skills
-III-6				NA	NA	NA	-NA
-IV-1				36	43	-8.2	-Mild intellectual disability, can speak few words, normal motor skills and self-care skills
-IV-3				33	43	-8.2	-Mild level mental retardation, normal motor skills, no fits, impaired speech
-IV-8				30	43	-8.2	-Mild mental retardation, can speak reluctantly, friendly behavior, no physical abnormality
**Fam. 3**	*CDK5RAP2*	NM_018249.5; c.448C>T;p.(Arg150*)	3				
-V-1				10	39	-10	-Developmental delay, mild level of intellectual disability, self-care skills, physically week, normal motor skills, speech impairment, short stature (-5 SD)
-V-2				8	37	-12	-Moderate intellectual disability, retarded growth, can speak few words, no history of seizures, reduced height (-5SD)
-V-3				5	35.5	-12	-Mild mental retardation, development delayed and impaired speech, hyperactive, short stature (-6 SD)

**Fam. 4**	*WDR62*	NM_001083961.1; c.2195C>T;p.(Thr732Ile)	5				
-V-4				08	38	-11	-Mild level of intellectual disability, speech impairment, delayed milestones
-V-6				18	38	-12.5	-Mild intellectual disability, short stature (-6 SD), normal motor skills
-VI-1				08	39	-9.5	-Mild mental retardation, imprecise articulation, normal motor skills, seizures
-VI-3				11	38	-10	-Mild intellectual disability, delayed milestones, normal motor skills
-VI-4				02	40.5	-7	-Moderate level of intellectual disability , impaired speech, strabismus
**Fam. 5**	*ASPM*	NM_018136.4; c.8508_8509delGA;p.(Lys 2837Metfs*34)	4				
-IV-1				NA	NA	NA	-Severe intellectual disability, aggressive behavior, indifferent to personal care
-IV-2				27	43	-8.5	-Mild intellectual disability, aggressive behavior, able to perform personal care
-IV-3				18	47	-6	-Mild intellectual disability, aggressive behavior, self-care skills
-V-1				9 months	33.5	-8	-Severe intellectual disability, aggressive behavior, indifferent to personal care
**Fam.6**	*ASPM*	NM_018136.4; c. 8190_8193delAGAA;p.(Ar g2732Lysfs*4)	7				
-IV-1				NA	NA	NA	-NA
-V-2				30	NA	NA	-NA
-V-3				17	38	-11.5	-Moderate level of intellectual disability, dumbfounded, aggressive and self-harming behavior
-V-9				NA	NA	NA	-NA
-VI-1				8	39	-10.5	-Mild intellectual disability, dumbfounded, self-care skills and normal motor skills
-VI-5				4	38	-11	-Moderate intellectual disability, dumbfounded, no self-care skills, aggressive behavior, self-injurious behavior
-VI-6				9 months	35	-9	-Normal health and early milestone, can sit and crawl, physically active
**Fam. 7**	*ASPM*	NM_018136.4; c.1260_1266delTCAAGT C;p.(Gln421Hisfs*32)	3				
-IV-1				NA	NA	NA	-Mild intellectual disability, self-care skills, imprecise articulation
-IV-2				34	39.5	-11.5	-Severe intellectual disability, hyperactive, can speak a few words, no self-care skills, aggressive

							behavior, self-injurious
-IV-6				30	38	-12.5	-Mild intellectual disability, self-care skills, imprecise articulation
**Fam. 8**	*WDR62*	NM_001083961.1; c.3936dupC;p.(Val1313Argfs*18)					
-IV-1			3	30	43	-10.7	-Moderate intellectual disability, no self-care skills, fits, aggressive behavior starts beating and scolding in anger
-IV-3				26	43	-8	-Mild mental retardation, self-care skills
-IV-7				22	45	-6.8	-mild mental disability, history of fits with bleeding from the mouth
**Fam. 9**	*WDR62*	NM_001083961.1; c.4241dupT;p.(Ser1415Glufs*40)					
-IV-3			7	NA	NA	NA	-NA
-V-2				NA	NA	NA	-NA
-V-3				NA	NA	NA	-NA
-V-4				NA	NA	NA	-NA
-V-5				NA	NA	NA	-NA
-V-7				29	43	-8	-Mild intellectual disability, aggressive behavior, history of seizure, one leg lost in an accident, no self-care skills, impaired speech
-V-10				8	38	-10	-Moderate level of intellectual disability, dumbfounded, aggressive behavior, self-punishing as well as beating others, no self-care skills
**Fam.10**	*WDR62*	NM_001083961.1; c.3361delG;p.(Ala1121Glnfs*6)					
-V-1			5	NA	NA	NA	-NA
-V-3				NA	NA	NA	-NA
-VI-1				16	45	-6.8	-Moderate level of intellectual disability, impaired speech, no self-care skills, aggressive behavior
-VI-5				7	43	-7	-Mild intellectual disability, dumbfounded, no self-care skills, normal motor skills
-VI-6				2	37	-9.8	-Mild intellectual disability, dumbfounded, no self-care skills, normal early milestones
**Fam. 11**	*CENPJ*	NM_018451.4; c.18delC;p.(Ser7Profs*2)					
-IV-1			3	NA	NA	NA	-NA
-VI-3				NA	NA	NA	-NA
-VI-4				18	44.5	-7.5	-Mild intellectual disability, dumbfounded, self-care skills, normal motor skills

**Fam. 12**<br>-IV-3<br><br>-IV-7	*ASPM*	NM_018136.4;<br>c.5149delA;p.(Ile1717*)	2	25<br><br>13	45<br><br>39	-8<br><br>-10	-Mild intellectual disability, impaired speech, self-care skills, normal motor skills<br>-Mild level of intellectual disability, physically active, can speak only a few words, aggressive behavior
**Fam.13**<br>-IV-1<br><br>-IV-2	*ASPM*	NM_018136.4;<br>c.1260_1266delTCAAGTC;p.(Gln421Hisfs*32)	2	4<br><br>6	36<br><br>38	-9<br>-11	-Mild level of intellectual disability, speech impairment<br>-Moderate intellectual disability, no self-care skills, aggressive behavior
**Fam.14**<br>-IV-1<br><br>-IV-2<br><br>-IV-3	*ASPM*	NM_018136.4;<br>c.1602_1605delTCAA;p.(Asn534Lysfs*14)	3	2<br><br>2<br><br>NA	40<br><br>41<br><br>NA	-9<br><br>-11<br><br>NA	-Moderate level of intellectual disability, dumbfounded, bilateral hearing impairment, small anterior fontanel was also observed<br>-Moderate intellectual disability, dumbfounded, bilateral hearing impairment, Small anterior fontanel was also noticed<br>-NA
**Fam.15**<br>-IV-1<br><br>-IV-2<br><br>-IV-3	*ASPM*	NM_018136.4;<br>c.9190C>T;p.(Arg3064*)	3	12<br><br>17<br><br>NA	42<br><br>44<br><br>NA	-7<br><br>-9<br><br>NA	-Mild intellectual disability, no hearing or vision impairment, normal self-care skills<br>-Mild level of intellectual disability without any other gross abnormality<br>-NA
**Fam. 16**<br>-IV-1<br>-IV-2<br><br>-IV-3	*ASPM*	NM_018136.4;<br>c.9961C>T;p.(Gln3321*)	3	3<br>9<br><br>NA	37<br>41<br><br>NA	-7<br>-10<br><br>NA	-Moderate intellectual disability, speech impairment.<br>-Moderate level of intellectual disability, dumbfounded without any other physical or facial anomaly<br>-NA
**Fam.17**<br>-V-1<br>-V-2<br><br>-V-3	*ASPM*	NM_018136.4;<br>c.9557C>G;p.(Ser3186*)	3	24<br>15<br><br>NA	38<br>41<br><br>NA	-10<br>-9.5<br><br>NA	-Severe intellectual disability, no self-care skills, stutter<br>-Severe intellectual disability, aggressive, no self-care skills<br>-NA

**Fam. 18**<br>-IV-1<br>-IV-2<br>-IV-3	*ASPM*	NM_018136.4;<br>c.4212G>A;p.(Trp1404*)	2	30<br>45<br>NA	40<br>41<br>NA	-10<br>-9<br>NA	-Mild level of intellectual disability, dumbfounded<br>-Moderate intellectual impairment and delayed speech<br>-NA
**Fam.19**<br>-IV-3<br>-IV-4<br>-IV-5<br>-V-1	*ASPM*	NM_018136.4;<br>c.5149delA;p.(Ile1717*fs)	4	2<br>16<br>NA<br>NA	41<br>45.5<br>NA<br>NA	-6<br>-7<br>NA<br>NA	-Moderate intellectual disability, self-care skills<br>-Moderate level of intellectual disability, normal self-care skills, cognitive impairment without any other physical abnormality<br>-NA<br>-NA
**Fam. 20**<br>-V-1	*ASPM*	NM_018136.4;<br>c.727C>T;p.(Arg243*)	1	11	38	-12	-Mild intellectual disability, indifferent to personal care, excessive salivation, aggressive behavior, speech impairment
**Fam. 21**<br>-IV-1<br>-IV-2	*WDR62*	NM_001083961.1;<br>c.1194G>A;p.(Trp398*)	2	15<br>24	48<br>44:5	-5<br>-5:5	-Severe intellectual disability, aggressive behavior, able to self-care without the ability of self-feeding.<br>-Seizures, moderate intellectual disability, speech impairment
**Fam. 22**<br>-IV-1	*ASPM*	NM_018136.4;<br>c.9601C>T;p.(Gln3201*)	1	4	35	-9	-Moderate intellectual disability, developmental delay, indifferent to personal care
**Fam. 23**<br>-IV-1	*CEP135*	NM_025009.4;<br>c.3157G>T;p.(Glu1053*)	1	27	48	-7	-Mild intellectual disability, personal-care skills, autistic-like behavior, strabismus with normal vision, precise speech
**Fam.24**<br>-IV-1<br>-IV-2<br>-IV-3	*STIL*	NM_001048166.1;<br>c.3694C>T;p.(Arg1232*)	3	15<br>11<br>13	45<br>44<br>44	-6<br>-4.66<br>-6.66	-Mild intellectual disability, able to perform personal care<br>-Mild intellectual disability, able to perform self-care functions<br>-Epileptic shocks started at the age of 10 years, aggressive behavior, speech impairment

**Fam.25**<br>-IV-1	*ASPM*	NM_018136.4; c.1615_1616delGA;p.(Glu539Argfs*15)	1	38	NA	NA	-Severe intellectual disability, indifferent to personal care
**Fam.26**<br>-IV-1<br>-IV-2	*ASPM*	NM_018136.4; c. 8718_8721delTTTA;p.(Leu2907Argfs*30)	2	35<br>31	45<br>42	-6<br>-8	-Severe intellectual disability, blurry vision, white spots on both hands, cup-shaped ears, imprecise articulation<br>-Severe intellectual disability, deafness, inability to walk, poliomyelitis in childhood
**Fam.27**<br>-V-1<br>-VI-1	*ASPM*	NM_018136.4; c.3193C>T;p.(Gln1065*)	2	NA<br>NA	NA<br>NA	NA<br>NA	-Severe intellectual disability, poor self-care skills<br>-Severe intellectual disability and speech impairment, no personal-care skills
**Fam.28**<br>IV-1<br>-IV-2<br>-IV-4	*ASPM*	NM_018136.4; c.9286C>T;p.(Arg3096*)	3	47<br>45<br>42	40<br>38<br>NA	-9.9<br>-9.7<br>NA	-Classical primary microcephaly, profound intellectual disability, aggressive behavior<br>-Microcephaly, poor self-care skills, severe intellectual disability<br>-Small head size, indifferent to personal care, aggressive behavior, severe intellectual disability
**Fam.29**<br>-V-1<br>-V-2	*STIL*	NM_001048166.1; c.3759dupT;p.(Pro1254Serfs*2)	2	12<br>5	42<br>42	-8<br>-8	-Mild intellectual disability, able to speak a few words, friendly behavior<br>-Mild intellectual disability, normal self-care skills, precise articulation without the ability to make a complete sentence
**Fam.30**<br>-IV-1<br>-IV-2<br>-IV-4<br>-IV-5	*ASPM*	NM_018136.4; c.5959C>T; p.(Gln1987*)	3	22<br>22<br>27<br>30	NA<br>NA<br>45<br>45	NA<br>NA<br>-5<br>-5.6	-NA<br>-NA<br>-Profound intellectual disability, aggressive behavior, excessive salivation<br>-Poor self-care skills, profound intellectual disability
**Fam. 31**<br>-III-1<br>-III-2<br>-III-3<br>-IV-4	*ASPM*	NM_018136.4; c.7782_7783delGA; p.(Lys2595Serfs*6)	4	NA<br>NA<br>27<br>17	NA<br>NA<br>NA<br>44	NA<br>NA<br>NA<br>-6.5	-NA<br>-NA<br>-NA<br>-Mild intellectual disability, precise speech, ability

							to perform self-care functions
**Fam. 32**<br>-II-3	*ASPM*	NM_018136.4;<br>c.6854_6855delTC<br>p.(Leu2285Glnfs*32)<br><br>NM_018136.4;<br>c.9976_9977dupGT<br>p.(Ser3327Tyrfs*14)<br><br>NM_018136.4;c.3741G>A<br>; p.(Lys1247=)	1	2.5	37	-9	-Normal early milestones, started walking at the age of 9 months

Abbreviations: NA, not available; OFC; Occipitofrontal Circumference; SD, standard deviation.

TABLE S4 List of disease causing DNA variants reported in *ASPM* after the report of Létard and colleagues (Letard et al., 2018).

cDNA mutation	Protein mutation	Origin	Disorder/ Zygosity	Reference
**c.727C>T**	**p.(Arg243*)**	**Pakistani/second cousin**	**PM/H**	**This Study (Family 20)**
c.1386delC	p.(Tyr462*)	Caucasian /non-consanguineous	PM/H	(Marakhonov et al., 2018)
c.1402_1406del[†]	p.(Asn468Serfs*2)	Korean/non-consanguineous	PM/CH	(Moriwaki et al., 2019)
**c.1602_1605delTCAA**	**p.(Asn534Lysfs*14)**	**Pakistani/first cousin**	**PM/H**	**This Study (Family 14)**
**c.1615_1616delGA**	**p.(Glu539Argfs*15)**	**Pakistani/first cousin**	**PM/H**	**This Study (Family 25)**
c.1952_1955delAAAC	p.(Asn653Lysfs*14)	Saudi	PM/H	(Shaheen et al., 2019)
c.2738dupT	p.(Cys914fs)	Iranian/consanguineous	PM/H	(Bazgir, Agha Gholizadeh, Sarvar, & Pakzad, 2019)
**c.3193C>T**	**p.(Gln1065*)**	**Pakistani/consanguineous**	**PM/H**	**This Study (Family 27)**
c.3384_3385dupT	p.(Lys1129*)	Indian/first cousin	PM/H	(Bhargav, Sreedevi, Swapna, Vivek, & Kovvali, 2017)
c.3796G>T	p.(Glu1266*)	Kosovan/non-consanguineous	PM/H	(Boonsawat et al., 2019)
c.5219_5225delGAGGATA	p.(Arg1740Thrfs*7)	Turkish/consanguineous	PM/H	(McSherry et al., 2018)
c.7543C>T	p.(Arg2515*)	Pakistani/consanguineous	PM/H	(A. Khan, Wang, Han, Ahmad, & Zhang, 2018)
c.7684A>G	p.(Ser2562Gly)			(R. Li et al., 2017)
c.7782_7783delGA[‡]<br>c.9742_9745delAAAC	p.(Lys2595Serfs*6)<br>p.(Lys3248Serfs*13)	Japanese/non-consanguineous	PM/H/CH	(Ahmed et al., 2019; Okamoto, Kohmoto, Naruto, Masuda, & Imoto, 2018)
**c.8718_8721delTTTA**	**p.(Leu2907Argfs*30)**	**Pakistani/first cousin**	**PM/H**	**This Study (Family 26)**

**c.9601C>T**	**p.(Gln3201*)**	**Pakistani/first cousin**	**PM/H**	**This Study (Family 22)**
**c.9961C>T**	**p.(Gln3321*)**	**Pakistani/first cousin**	**PM/H**	**This Study (Family 16)**
**c.6854_6855delTC**<br>**c.9976_9977dupGT**<br>**c.3741G>A**	**p.(Leu2285Glnfs*32)**<br>**p.(Ser3327Tyrfs*14)**<br>**p.(Lys1247=)**	**Pakistani/non-consanguineous**	**PM/CH**	**This Study (Family 32)**
c.10013delA	p.(Asp3338Valfs*2)	Pakistani/first cousin	PM/H	(M. A. Khan et al., 2017)

Note: PM stands for primary microcephaly, **H** for homozygous and **CH** for compound heterozygous. Mutation report of this study is mentioned in bold. †This mutation was reported in the form of compound heterozygous form along with a known mutation, c.9697C > T, p.(Arg3233*)). ‡Recently this mutation was found in a Pakistani family with homozygous state.

TABLE S5 List of disease causing DNA variants reported in *WDR62*

cDNA mutation	Protein mutation	Origin	Disorder/zygosity	Reference
c.28G>T<br>c.189G>T	p.(Ala10Ser)<br>p.(Glu63Asp)	Chinese/consanguineous	PM/CH	(Banerjee et al., 2016)
c.193G>A	p.(Val65Met)	Pakistani/consanguineous<br>Saudi/first cousin	PM/H	(Nicholas et al., 2010; Yu et al., 2010)
c.332G>C	p.(Arg111Thr)	Pakistani/first cousin	PM/H	(Sajid Hussain et al., 2013)
c.363delT	p.(Asp112Metfs*5)	Mexican/first cousin	PM/H	(Yu et al., 2010)
c.390G>A	p.(Glu130Glu)	Sudanese/consanguineous	PM/H	(Bastaki et al., 2016)
c.535_536insA	p. (Met179fs*21)	India/consanguineous	PM/H	(Bhat et al., 2011)

c.900C>A	p.(Cys300*)			
c.668T>C	p.(Phe223Ser)	Romani/consanguineous<br>Romani/non-consanguineous	PM with abnormal cortical architecture/H	(Zombor et al., 2019)
c.671G>C	p.(Trp224Ser)	Turkish/first cousin	PM/H	(Bilguvar et al., 2010)
c.731C>T<br>c.2413G>T	p. (Ser244Leu)<br>p.(Glu805*)	Japanese/non-consanguineous	PM/CH	(Miyamoto et al., 2017)
c.797C>T<br>c.1102G>A	p.(Ala266Val)<br>p.(Asp368Asn)	Saudi/consanguineous	PM/CH	(Naseer et al., 2019)
c.1027C>T	p.(Gln343*)	Moroccan/consanguineous	PM/H	(Jaouad et al., 2018)
c.883-4_890del<br>c.1684C>G	p.His562Asp	South Korean/ non-consanguineous	PM/CH	(Yi et al., 2019)
c.883-1273_ 1237-850del	Microdeletion of exon 8 and 9	Pakistani/consanguineous	PM/H	(Wang, Khan, Han, & Zhang, 2017)
c.1043+1G>A	p.Ser348Argfs*63	Turkish/first cousin	PM/H	(Yu et al., 2010)
c.1143delA	p.(His381Profs*48)	Pakistani/consanguineous	PM/H	(Memon et al., 2013)
c.1194G>A	p.(Trp398*)	Pakistani/first cousin	PM/H	(Sajid Hussain et al., 2013)
c.1198G>A	p.(Glu400Lys)	Hispanic/consanguineous	PM/H	(Bacino, Arriola, Wiszniewska, & Bonnen, 2012)
c.1313G>A	p.(Arg438His)	Pakistani/consanguineous	PM/H	(Kousar et al., 2011; Nicholas et al., 2010)
c.1313G>A<br>c.2864_2867delACAG	p.(Arg438His)<br>p.(Asp955Alafs*112)	German /non-consanguineous	PM/CH	(Farag et al., 2013)
c.1408C>T	p.(Gln470*)	Turkish/first cousin	PM/H	(Bilguvar et al., 2010)
c.1531G>A	p.(Asp511Asn)	Pakistani/consanguineous	PM/H	(Kousar et al., 2011;

				Nicholas et al., 2010)
c.1576G >T<br>c.1576G >A	p.(Glu526*)<br>p.(Glu526Lys)	Turkish/first cousin	PM/H	(Bilguvar et al., 2010)
c.1605dupT[†]<br>Duplication of 17q25-qter,<br>*TBCD*;c.3361T>G[¶]	p.(Glu536*)<br><br>p.Phe1121Val	Turkish/consanguineous	PM/H	(Poulton et al., 2014)
c.1821dupT	p.(Arg608Serfs*26)	French Canadian/first cousin	PM/H	(McDonell et al., 2014)
c.1942C>T	p.(Gln648*)	Pakistani/first cousin	PM/H	(Kousar et al., 2011)
c.1973_1974del	p.Val658Glufs*14	Swedish/ consanguineous	PM/H	(Kvarnung et al., 2018)
c.2115C>G	p.(Gly705Gly)	Iranian/consanguineous	PM, cerebral atrophy/H	(Najmabadi et al., 2011)
**c.2195C>T**	**p.(Thr732Ile)**	**Pakistani/ consanguineous**	**PM/H**	**This study (Family 4)**
c.2083delA<br>c.2472_2473delAG	p.(Ser696Alafs*4)<br>p.(Gln918Glyfs*18)	Unknown/non-consanguineous	Polymicrogyria /CH	(Murdock et al., 2011)
c.2520+5G>T	p.(Asp823Alafs*5)	Pakistani/consanguineous	PM/H	(Wang et al., 2017)
c.2527dupG	p.(Asp843Glyfs*3)	Pakistani/first cousin	PM/H	(Rupp, Rauf, Naveed, Windpassinger, & Mir, 2014)
c.2588G>A	p.(Arg863His)	Tunisian/consanguineous	PM/H	(Poulton et al., 2014)
c.2667_2668GA>TT	p.(Met889Ile)<br>p.(Lys890*)	Pakistani/consanguineous	PM/H	(Wang et al., 2017)
c.2863delGACA	p.(Asp955Alafs*112)	Turkish/second cousin	PM/H	(Poulton et al., 2014; Sgourdou et al., 2017)
c.2867+4_ 2867+7delGGTG	p.(Ser956Cysfs*38)	Turkish/first cousin	PM/H	(Yu et al., 2010)

c.3012_3034dup	p.(Pro1012fs)	Unknown	PM (with or without cortical malformations)/unknown	National Center for Biotechnology Information. ClinVar; [VCV000437292.1]
c.3232G>A	p.(Ala1078Thr)	Pakistani/consanguineous	PM/H	(Nicholas et al., 2010)
c.3335+1G>C		Italian /non-consanguineous	PM/H	(Nardello et al., 2018)
c.3361delG	p.(Ala1121Glnfs*6)	Pakistani/first cousin	PM/H	(Sajid Hussain et al., 2013)
c.3406C>T	p.(Arg1136*)	Unknown	ID and global developmental delay/H	(McSherry et al., 2018)
c.3503G>A	p.(Trp1168*)	Pakistani/second cousin	PM/H	(Sajid Hussain et al., 2013)
c.3839_ 3855delGCCAAGAGCCTGCCCTG	p.(Gly1280Alafs*21)	Turkish/first cousin	PM/H	(Bilguvar et al., 2010; Yu et al., 2010)
c.3878C>A	p.(Ala1293Asp)	Saudi/consanguineous	PM/H	(Naseer et al., 2017)
c.3936dupC[‡]	p. (Val1314Argfs*18)	Pakistani/consanguineous<br>Turkish/first cousin<br>Unknown	PM/H	(Kousar et al., 2011; Nicholas et al., 2010; Yu et al., 2010)
c.4205_ 4208delTGCC	p.(Val1402Glyfs*12)	Turkish/first cousin	PM/H	(Bilguvar et al., 2010)
c.4241dupT	p.(Leu1414Leufs*41)	Pakistani/unknown	PM/H	(Nicholas et al., 2010)

Note: PM stands for primary microcephaly, **H** for homozygous and **CH** for compound heterozygous. [†]This mutation is the corrected form of c.1605_1606insT which is modified according to the HGVS (Human Genome Variation Society) guidelines. [‡]Renamed (c.3935_ 3936insT) according to the HGVS recommendations. [¶]Indicates modifiers where duplication of chromosomal segment 17q25-qter on one allele and a missense mutation, c.3361T>G;p.(Phe1121Val), of *TBCD* on the other, non-duplicated allele was found.

TABLE S6 List of disease causing DNA variants identified in *CDK5RAP2*

cDNA mutation	Protein mutation	Origin	Disorder/Zygosity	Reference
c.246T>A	p.(Tyr82*)	Pakistani/first cousin	PM/H	(Bond et al., 2005; Hassan et al., 2007)
c.4005-15A>G		Northern Pakistan/first cousins	PM/H	(Bond et al., 2005)
**c.448C>T**	**p.(Arg150*)**	**Pakistani /consanguineous**	**PM**/H	**This study (Family 3)**
c.700G>T	p.(Glu234*)	Somali/second cousin	PM/H	(Pagnamenta et al., 2012)
c.4441C>T	p.(Arg1481*)	Italian/third cousins	PM/H	(Issa et al., 2013)
c.4546G>T<br>c.4672C>T	p.(Glu1516*)<br>p.(Arg1558*)		PM/ CH	(Boonsawat et al., 2019; Pagnamenta et al., 2012)
c.524_528del<br>c.4005-1G>A		Caucasian/non-consanguinity	PM/ CH	(Issa et al., 2013; Tan et al., 2014)
c.4441C>T<br>c.5227C>T	p.(Arg1481*)<br>p.(Gln1743*)	Guatemalan and Honduran/non-consanguinity	PM/ CH	(M. H. Li et al., 2015)
c. 4005-9A>G	p.(Arg1335Serfs*3)	Turkish /second cousin	SS/H	(Yigit et al., 2015)
c.383+1G>C,	p.(Lys129*)	Pakistani /unknown		
c.4187T>C,<br>Digenic with mutation of *CEP152;*c.3014_3015delAAinsT	p.(Met1396Thr)<br>p.(Lys1005Ilefs*16)	German /non-consanguineous	SS/CH	
c.4604+1G>C<br>c.3097delG	p.(Val1033fs*41)	British Caucasian/unknown	PM/ CH	(Pagnamenta et al., 2016)
c.1279C>T	p.(Arg427*)	Pakistani/first cousin	PM/H	(Ahmad et al., 2017)
c.4114C >T	p.(Arg1372*)	Pakistani/first cousins	PM/H	(Sukumaran et al., 2017)
c.280G>A<br>c.3695A>G	p.(Gly94Arg)<br>p.(Asn1232Ser)	French Canadian /non-consanguineous	ACC/CH	(Jouan et al., 2016)

c.5127_5128dupGT	p.(Ser1710Cysfs*22)	Pakistani/consanguineous		(Abdullah et al., 2017)
c.4055A>G	p.(Glu1352Gly)	Saudi/consanguineous	PM/H	(Alfares et al., 2018)
c.4672C>T	p.(Arg1558*)	Saudi/consanguineous	PM/H	(Alfares et al., 2018)
c.558_559delGA<br>c.4441C>T	p.(Glu186Aspfs*32)<br>p.(Arg1481*)		PM /CH	(Boonsawat et al., 2019; Issa et al., 2013)
c.3928G>T<br>c.4546G>T	p.(Glu1310*)<br>p.(Glu1516*)	Unknown	PM+short stature/CH	(Boonsawat et al., 2019; Lancaster et al., 2013)
c.140delT,<br>c.384_2106del	p.(Val47Glyfs*14)<br>p.(Lys129Phefs*22)	Saudi/non-consanguineous	PM/CH	(Shaheen et al., 2019)
c.4005-15A>G,<br>Digenic with *CNTRL*<br>c.586C>T	p.(Arg196*)	Saudi/second cousin	PD/ CH	(Shaheen et al., 2019)

Note: PM stands for primary microcephaly, **PD** for primordial dwarfism, **ACC** for agenesis of the corpus callosum, **SS** for Seckel syndrome, **H** for homozygous and **CH** for compound heterozygous. Mutation in bold is the finding of this study.

TABLE S7 List of disease causing variants identified in *CENPJ*

cDNA mutation	Protein mutation	Origin	Disorder/Zygosity	Reference
**c.18delC**	**p.(Ser7Profs*2)**	**Pakistani/ consanguineous**	**PM/H**	**(Bond et al., 2005; Sajid Hussain et al., 2013), This study (Family 11)**
c.3704A>T	p.(GLu1235Val)	Brazilian/first cousin	PM/H	(Bond et al., 2005)
c.3242_3246delTCAG	p.(Ser1081Argfs*8)	Pakistani/first cousin	PM/H	(Gul et al., 2006)
c.2462C>T	p.(Thr821Met)	Iranian/unknown	PM/H	(Darvish et al., 2010)
c.3302-1G>C			SS/H	(Al-Dosari, Shaheen, Colak, & Alkuraya, 2010)

†[(3302_3366del), (3217_3366del), (3217_3477del)]	p.(1102_1122delfs*6), p.(1073_1122de), p.(1073_1159del)	Saudi/first cousin		
c.3302-1G>C, c.43_44del, c.133C>T, c.125_126delAA,	p.(Asn15Leufs*42), p.(Arg45*), p.(Lys42Argfs*15)	Saudi/non consanguineous Saudi/first Cousin Saudi/first Cousin	PM/H	(Shaheen et al., 2019)

Note: **PM** stands for primary microcephaly, **SS** for Seckel syndrome, **H** for homozygous and **CH** for compound heterozygous. †Three different transcripts produced due to the splice variant, c.3302-1G>C. Mutation in bold is the finding of this study.

TABLE S8 List of disease causing DNA variants identified in *STIL*

cDNA mutation	Protein mutation	Origin	Disorder/Zygosity	Reference
c.453+5G>A	p.(Asp89Glyfs*8)	Pakistani/first cousin	PM/H	(Kakar et al., 2015)
c.3715C>T	p.(Gln1240*)	Indian/consanguineous	PM+ developmental delay/H	(Kumar et al., 2009)
c.3655delG	P.(Leu1219*)			
c. 2829+1G>A	p.(Gly944Valfs*15)			
c.2392T>G	p.(Leu798Trp*)	Iranian/ consanguineous	PM/H	(Papari et al., 2013)
c.2354_2355dupGA c.3835C>T	p.(Lys786Glufs*5) (paternal) p.(Arg1280Cys) (maternal)	Unknown/Non-consanguineous	PM/CH	(Bennett et al., 2014)
**c.3694C>T**	**p.(Arg1232*)**	**Pakistani/ consanguineous**	**PM/H**	**This study (Family 24)**
**c.3759dupT**	**p.(Pro1254Serfs*2)**	**Pakistani/ consanguineous**	**PM/H**	**This study (Family 29)**

c.2150G>A	p.(Gly717Glu)	Turkish/ first cousin	Holoprosencephaly+ PM/H	(Mouden et al., 2015)
c.1231C>G<br>c.3370A>G	p.(His411Asp)<br>p.(Met1124Val)	Caucasian /non-consanguineous	Severe fetal microcephaly/CH	(Cristofoli, De Keersmaecker, De Catte, Vermeesch, & Van Esch, 2017)
c.3552_3553del	p.(Cys1184*)	Saudi/consanguineous	PM/H	(Shaheen et al., 2019)

Note: PM stands for primary microcephaly, **H** for homozygous and **CH** for compound heterozygous. In bold are findings reported in this study.

TABLE S9 List of disease causing DNA variants identified in *CEP135*

**cDNA mutation**	**Protein mutation**	**Origin**	**Disorder**	**Reference**
c.970delC	p.(Gln324Serfs*)	Pakistani/first cousin	PM/H	(Hussain et al., 2012)
c.1473+1G>A	p.(Glu417Glyfs*2)	Pakistani/first cousin	PM/H	(Farooq et al., 2016)
c.2722C>T	p.(Arg908*)	Saudi/first cousin	PD/H	(Shaheen et al., 2019)
**c.3157G>T**	**p.(Glu1053*)**	**Pakistani/consanguineous**	**PM/H**	**This study (Family 23)**

Note: PM stands for primary microcephaly, **PD** for primordial dwarfism, **H** for homozygous and **CH** for compound heterozygous. Mutation report of this study is mentioned in bold.

REFERENCES

Abdullah, U., Farooq, M., Mang, Y., Marriam Bakhtiar, S., Fatima, A., Hansen, L., . . . Mahmood Baig, S. (2017). A novel mutation in CDK5RAP2 gene causes primary microcephaly with speech impairment and sparse eyebrows in a consanguineous Pakistani family. *Eur J Med Genet, 60*(12), 627-630. doi:10.1016/j.ejmg.2017.07.017

Ahmad, I., Baig, S. M., Abdulkareem, A. R., Hussain, M. S., Sur, I., Toliat, M. R., . . . Nurnberg, P. (2017). Genetic heterogeneity in Pakistani microcephaly families revisited. *Clin Genet, 92*(1), 62-68. doi:10.1111/cge.12955

Ahmed, J., Windpassinger, C., Salim, M., Wiener, M., Petek, E., Schaflinger, E., . . . Khan, M. A. (2019). Genetic study of Khyber-Pukhtunkhwa resident Pakistani families presenting primary microcephaly with intellectual disability. *J Pak Med Assoc, 69*(12), 1812-1816. doi:10.5455/JPMA.300681

Al-Dosari, M. S., Shaheen, R., Colak, D., & Alkuraya, F. S. (2010). Novel CENPJ mutation causes Seckel syndrome. *J Med Genet, 47*(6), 411-414. doi:10.1136/jmg.2009.076646

Alfares, A., Alhufayti, I., Alsubaie, L., Alowain, M., Almass, R., Alfadhel, M., . . . Eyaid, W. (2018). A new association between CDK5RAP2 microcephaly and congenital cataracts. *Ann Hum Genet, 82*(3), 165-170. doi:10.1111/ahg.12232

Awad, S., Al-Dosari, M. S., Al-Yacoub, N., Colak, D., Salih, M. A., Alkuraya, F. S., & Poizat, C. (2013). Mutation in PHC1 implicates chromatin remodeling in primary microcephaly pathogenesis. *Hum Mol Genet, 22*(11), 2200-2213. doi:10.1093/hmg/ddt072

Bacino, C. A., Arriola, L. A., Wiszniewska, J., & Bonnen, P. E. (2012). WDR62 missense mutation in a consanguineous family with primary microcephaly. *Am J Med Genet A, 158A*(3), 622-625. doi:10.1002/ajmg.a.34417

Banerjee, S., Chen, H., Huang, H., Wu, J., Yang, Z., Deng, W., . . . Li, X. (2016). Novel mutations c.28G>T (p.Ala10Ser) and c.189G>T (p.Glu63Asp) in WDR62 associated with early onset acanthosis and hyperkeratosis in a patient with autosomal recessive microcephaly type 2. *Oncotarget, 7*(48), 78363-78371. doi:10.18632/oncotarget.13279

Basit, S., Al-Harbi, K. M., Alhijji, S. A., Albalawi, A. M., Alharby, E., Eldardear, A., & Samman, M. I. (2016). CIT, a gene involved in neurogenic cytokinesis, is mutated in human primary microcephaly. *Hum Genet, 135*(10), 1199-1207. doi:10.1007/s00439-016-1724-0

Bastaki, F., Mohamed, M., Nair, P., Saif, F., Tawfiq, N., Aithala, G., . . . Hamzeh, A. R. (2016). Novel splice-site mutation in WDR62 revealed by whole-exome sequencing in a Sudanese family with primary microcephaly. *Congenit Anom (Kyoto), 56*(3), 135-137. doi:10.1111/cga.12144

Bazgir, A., Agha Gholizadeh, M., Sarvar, F., & Pakzad, Z. (2019). A Novel Frameshift Mutation in Abnormal Spindle-Like Microcephaly (ASPM) Gene in an Iranian Patient with Primary Microcephaly: A Case Report. *Iran J Public Health, 48*(11), 2074-2078. Retrieved from http://www.ncbi.nlm.nih.gov/pubmed/31970108

Bennett, H., Presti, A., Adams, D., Rios, J., Benito, C., & Cohen, D. (2014). A prenatal presentation of severe microcephaly and brain anomalies in a patient with novel compound heterozygous mutations in the STIL gene found postnatally with exome analysis. *Pediatr Neurol, 51*(3), 434-436. doi:10.1016/j.pediatrneurol.2014.05.023

Bhargav, D. S., Sreedevi, N., Swapna, N., Vivek, S., & Kovvali, S. (2017). Whole exome sequencing identifies a novel homozygous frameshift mutation in the ASPM gene, which causes microcephaly 5, primary, autosomal recessive. *F1000Res, 6*, 2163. doi:10.12688/f1000research.12102.1

Bhat, V., Girimaji, S. C., Mohan, G., Arvinda, H. R., Singhmar, P., Duvvari, M. R., & Kumar, A. (2011). Mutations in WDR62, encoding a centrosomal and nuclear protein, in Indian primary microcephaly families with cortical malformations. *Clin Genet, 80*(6), 532-540. doi:10.1111/j.1399-0004.2011.01686.x

Bilguvar, K., Ozturk, A. K., Louvi, A., Kwan, K. Y., Choi, M., Tatli, B., . . . Gunel, M. (2010). Whole-exome sequencing identifies recessive WDR62 mutations in severe brain malformations. *Nature, 467*(7312), 207-210. doi:10.1038/nature09327

Bond, J., Roberts, E., Springell, K., Lizarraga, S. B., Scott, S., Higgins, J., . . . Woods, C. G. (2005). A centrosomal mechanism involving CDK5RAP2 and CENPJ controls brain size. *Nat Genet, 37*(4), 353-355. doi:10.1038/ng1539

Boonsawat, P., Joset, P., Steindl, K., Oneda, B., Gogoll, L., Azzarello-Burri, S., . . . Rauch, A. (2019). Elucidation of the phenotypic spectrum and genetic landscape in primary and secondary microcephaly. *Genet Med, 21*(9), 2043-2058. doi:10.1038/s41436-019-0464-7

Braun, D. A., Lovric, S., Schapiro, D., Schneider, R., Marquez, J., Asif, M., . . . Hildebrandt, F. (2018). Mutations in multiple components of the nuclear pore complex cause nephrotic syndrome. *J Clin Invest, 128*(10), 4313-4328. doi:10.1172/JCI98688

Cristofoli, F., De Keersmaecker, B., De Catte, L., Vermeesch, J. R., & Van Esch, H. (2017). Novel STIL Compound Heterozygous Mutations Cause Severe Fetal Microcephaly and Centriolar Lengthening. *Mol Syndromol, 8*(6), 282-293. doi:10.1159/000479666

Darvish, H., Esmaeeli-Nieh, S., Monajemi, G. B., Mohseni, M., Ghasemi-Firouzabadi, S., Abedini, S. S., . . . Najmabadi, H. (2010). A clinical and molecular genetic study of 112 Iranian families with primary microcephaly. *J Med Genet, 47*(12), 823-828. doi:10.1136/jmg.2009.076398

DiStasio, A., Driver, A., Sund, K., Donlin, M., Muraleedharan, R. M., Pooya, S., . . . Stottmann, R. W. (2017). Copb2 is essential for embryogenesis and hypomorphic mutations cause human microcephaly. *Hum Mol Genet, 26*(24), 4836-4848. doi:10.1093/hmg/ddx362

Farag, H. G., Froehler, S., Oexle, K., Ravindran, E., Schindler, D., Staab, T., . . . Kaindl, A. M. (2013). Abnormal centrosome and spindle morphology in a patient with autosomal recessive primary microcephaly type 2 due to compound heterozygous WDR62 gene mutation. *Orphanet J Rare Dis, 8*, 178. doi:10.1186/1750-1172-8-178

Farooq, M., Fatima, A., Mang, Y., Hansen, L., Kjaer, K. W., Baig, S. M., . . . Tommerup, N. (2016). A novel splice site mutation in CEP135 is associated with primary microcephaly in a Pakistani family. *J Hum Genet, 61*(3), 271-273. doi:10.1038/jhg.2015.138

Genin, A., Desir, J., Lambert, N., Biervliet, M., Van Der Aa, N., Pierquin, G., . . . Abramowicz, M. (2012). Kinetochore KMN network gene CASC5 mutated in primary microcephaly. *Hum Mol Genet, 21*(24), 5306-5317. doi:10.1093/hmg/dds386

Guemez-Gamboa, A., Nguyen, L. N., Yang, H., Zaki, M. S., Kara, M., Ben-Omran, T., . . . Gleeson, J. G. (2015). Inactivating mutations in MFSD2A, required for omega-3 fatty acid transport in brain, cause a lethal microcephaly syndrome. *Nat Genet, 47*(7), 809-813. doi:10.1038/ng.3311

Guernsey, D. L., Jiang, H., Hussin, J., Arnold, M., Bouyakdan, K., Perry, S., . . . Samuels, M. E. (2010). Mutations in centrosomal protein CEP152 in primary microcephaly families linked to MCPH4. *Am J Hum Genet, 87*(1), 40-51. doi:10.1016/j.ajhg.2010.06.003

Gul, A., Hassan, M. J., Hussain, S., Raza, S. I., Chishti, M. S., & Ahmad, W. (2006). A novel deletion mutation in CENPJ gene in a Pakistani family with autosomal recessive primary microcephaly. *J Hum Genet, 51*(9), 760-764. doi:10.1007/s10038-006-0017-1

Hassan, M. J., Khurshid, M., Azeem, Z., John, P., Ali, G., Chishti, M. S., & Ahmad, W. (2007). Previously described sequence variant in CDK5RAP2 gene in a Pakistani family with autosomal recessive primary microcephaly. *Bmc Medical Genetics, 8*, 58. doi:10.1186/1471-2350-8-58

Hussain, M. S., Baig, S. M., Neumann, S., Nurnberg, G., Farooq, M., Ahmad, I., . . . Nurnberg, P. (2012). A truncating mutation of CEP135 causes primary microcephaly and disturbed centrosomal function. *Am J Hum Genet, 90*(5), 871-878. doi:10.1016/j.ajhg.2012.03.016

Hussain, M. S., Baig, S. M., Neumann, S., Peche, V. S., Szczepanski, S., Nurnberg, G., . . . Noegel, A. A. (2013). CDK6 associates with the centrosome during mitosis and is mutated in a large Pakistani family with primary microcephaly. *Hum Mol Genet, 22*(25), 5199-5214. doi:10.1093/hmg/ddt374

Issa, L., Kraemer, N., Rickert, C. H., Sifringer, M., Ninnemann, O., Stoltenburg-Didinger, G., & Kaindl, A. M. (2013). CDK5RAP2 expression during murine and human brain development correlates with pathology in primary autosomal recessive microcephaly. *Cereb Cortex, 23*(9), 2245-2260. doi:10.1093/cercor/bhs212

Jackson, A. P., Eastwood, H., Bell, S. M., Adu, J., Toomes, C., Carr, I. M., . . . Woods, C. G. (2002). Identification of microcephalin, a protein implicated in determining the size of the human brain. *Am J Hum Genet, 71*(1), 136-142. doi:10.1086/341283

Jaouad, I. C., Zrhidri, A., Jdioui, W., Lyahyai, J., Raymond, L., Egea, G., . . . Sefiani, A. (2018). A novel non sense mutation in WDR62 causes autosomal recessive primary microcephaly: a case report. *Bmc Medical Genetics, 19*. doi:Artn 11810.1186/S12881-018-0625-6Jeffers, L. J., Coull, B. J., Stack, S. J., & Morrison, C. G. (2008). Distinct BRCT domains in Mcph1/Brit1 mediate ionizing radiation-induced focus formation and centrosomal localization. *Oncogene, 27*(1), 139-144. doi:10.1038/sj.onc.1210595

Jouan, L., Ouled Amar Bencheikh, B., Daoud, H., Dionne-Laporte, A., Dobrzeniecka, S., Spiegelman, D., . . . Rouleau, G. A. (2016). Exome sequencing identifies recessive CDK5RAP2 variants in patients with isolated agenesis of corpus callosum. *Eur J Hum Genet, 24*(4), 607-610. doi:10.1038/ejhg.2015.156

Kadir, R., Harel, T., Markus, B., Perez, Y., Bakhrat, A., Cohen, I., . . . Birk, O. S. (2016). ALFY-Controlled DVL3 Autophagy Regulates Wnt Signaling, Determining Human Brain Size. *PLoS Genet, 12*(3), e1005919. doi:10.1371/journal.pgen.1005919

Kakar, N., Ahmad, J., Morris-Rosendahl, D. J., Altmuller, J., Friedrich, K., Barbi, G., . . . Borck, G. (2015). STIL mutation causes autosomal recessive microcephalic lobar holoprosencephaly. *Hum Genet, 134*(1), 45-51. doi:10.1007/s00439-014-1487-4

Khan, A., Wang, R., Han, S., Ahmad, W., & Zhang, X. (2018). Identification of a Novel Nonsense ASPM Mutation in a Large Consanguineous Pakistani Family Using Targeted Next-Generation Sequencing. *Genet Test Mol Biomarkers, 22*(3), 159-164. doi:10.1089/gtmb.2017.0229

Khan, M. A., Windpassinger, C., Ali, M. Z., Zubair, M., Gul, H., Abbas, S., . . . Nawaz, Z. (2017). Molecular genetic analysis of consanguineous families with primary microcephaly identified pathogenic variants in the ASPM gene. *J Genet, 96*(2), 383-387. Retrieved from http://www.ncbi.nlm.nih.gov/pubmed/28674240

Kousar, R., Hassan, M. J., Khan, B., Basit, S., Mahmood, S., Mir, A., . . . Ansar, M. (2011). Mutations in WDR62 gene in Pakistani families with autosomal recessive primary microcephaly. *BMC Neurol, 11*, 119. doi:10.1186/1471-2377-11-119

Kumar, A., Girimaji, S. C., Duvvari, M. R., & Blanton, S. H. (2009). Mutations in STIL, encoding a pericentriolar and centrosomal protein, cause primary microcephaly. *Am J Hum Genet, 84*(2), 286-290. doi:10.1016/j.ajhg.2009.01.017

Kvarnung, M., Taylan, F., Nilsson, D., Anderlid, B. M., Malmgren, H., Lagerstedt-Robinson, K., . . . Lundberg, E. S. (2018). Genomic screening in rare disorders: New mutations and phenotypes, highlighting ALG14 as a novel cause of severe intellectual disability. *Clin Genet, 94*(6), 528-537. doi:10.1111/cge.13448

Lancaster, M. A., Renner, M., Martin, C. A., Wenzel, D., Bicknell, L. S., Hurles, M. E., . . . Knoblich, J. A. (2013). Cerebral organoids model human brain development and microcephaly. *Nature, 501*(7467), 373-+. doi:Doi 10.1038/Nature12517

Li, M. H., Arndt, K., Das, S., Weiss, E. M., Wu, Y., Gwal, K., . . . Zackai, E. H. (2015). Compound heterozygote CDK5RAP2 mutations in a Guatemalan/Honduran child with autosomal recessive primary microcephaly, failure to thrive and speech delay. *American Journal of Medical Genetics Part A, 167*(6), 1414-1417.

Li, R., Sun, L., Fang, A., Li, P., Wu, Q., & Wang, X. (2017). Recapitulating cortical development with organoid culture in vitro and modeling abnormal spindle-like (ASPM related primary) microcephaly disease. *Protein Cell, 8*(11), 823-833. doi:10.1007/s13238-017-0479-2

Marakhonov, A. V., Konovalov, F. A., Makaov, A. K., Vasilyeva, T. A., Kadyshev, V. V., Galkina, V. A., . . . Zinchenko, R. A. (2018). Primary microcephaly case from the Karachay-Cherkess Republic poses an additional support for microcephaly and Seckel syndrome spectrum disorders. *BMC Med Genomics, 11*(Suppl 1), 8. doi:10.1186/s12920-018-0326-1

Martin, C. A., Murray, J. E., Carroll, P., Leitch, A., Mackenzie, K. J., Halachev, M., . . . Jackson, A. P. (2016). Mutations in genes encoding condensin complex proteins cause microcephaly through decatenation failure at mitosis. *Genes Dev, 30*(19), 2158-2172. doi:10.1101/gad.286351.116

McDonell, L. M., Warman Chardon, J., Schwartzentruber, J., Foster, D., Beaulieu, C. L., Consortium, F. C., . . . Boycott, K. M. (2014). The utility of exome sequencing for genetic diagnosis in a familial microcephaly epilepsy syndrome. *BMC Neurol, 14*, 22. doi:10.1186/1471-2377-14-22

McSherry, M., Masih, K. E., Elcioglu, N. H., Celik, P., Balci, O., Cengiz, F. B., . . . Tekin, M. (2018). Identification of candidate gene FAM183A and novel pathogenic variants in known genes: High genetic heterogeneity for autosomal recessive intellectual disability. *PLoS One, 13*(11), e0208324. doi:10.1371/journal.pone.0208324

Memon, M. M., Raza, S. I., Basit, S., Kousar, R., Ahmad, W., & Ansar, M. (2013). A novel WDR62 mutation causes primary microcephaly in a Pakistani family. *Mol Biol Rep, 40*(1), 591-595. doi:10.1007/s11033-012-2097-7

Mirzaa, G. M., Vitre, B., Carpenter, G., Abramowicz, I., Gleeson, J. G., Paciorkowski, A. R., . . . O'Driscoll, M. (2014). Mutations in CENPE define a novel kinetochore-centromeric mechanism for microcephalic primordial dwarfism. *Hum Genet, 133*(8), 1023-1039. doi:10.1007/s00439-014-1443-3

Miyamoto, T., Akutsu, S. N., Fukumitsu, A., Morino, H., Masatsuna, Y., Hosoba, K., . . . Matsuura, S. (2017). PLK1-mediated phosphorylation of WDR62/MCPH2 ensures proper mitotic spindle orientation. *Hum Mol Genet, 26*(22), 4429-4440. doi:10.1093/hmg/ddx330

Moawia, A., Shaheen, R., Rasool, S., Waseem, S. S., Ewida, N., Budde, B., . . . Hussain, M. S. (2017). Mutations of KIF14 cause primary microcephaly by impairing cytokinesis. *Ann Neurol, 82*(4), 562-577. doi:10.1002/ana.25044

Moriwaki, T., Yamazaki, N., So, T., Kosuga, M., Miyazaki, O., Narumi-Kishimoto, Y., . . . Fukuhara, Y. (2019). Normal early development in siblings with novel compound heterozygous variants in ASPM. *Hum Genome Var, 6*, 56. doi:10.1038/s41439-019-0088-0

Mouden, C., de Tayrac, M., Dubourg, C., Rose, S., Carre, W., Hamdi-Roze, H., . . . David, V. (2015). Homozygous STIL mutation causes holoprosencephaly and microcephaly in two siblings. *PLoS One, 10*(2), e0117418. doi:10.1371/journal.pone.0117418

Murdock, D. R., Clark, G. D., Bainbridge, M. N., Newsham, I., Wu, Y. Q., Muzny, D. M., . . . Ramocki, M. B. (2011). Whole-exome sequencing identifies compound heterozygous mutations in WDR62 in siblings with recurrent polymicrogyria. *Am J Med Genet A, 155A*(9), 2071-2077. doi:10.1002/ajmg.a.34165

Najmabadi, H., Hu, H., Garshasbi, M., Zemojtel, T., Abedini, S. S., Chen, W., . . . Ropers, H. H. (2011). Deep sequencing reveals 50 novel genes for recessive cognitive disorders. *Nature, 478*(7367), 57-63. doi:10.1038/nature10423

Nardello, R., Fontana, A., Antona, V., Beninati, A., Mangano, G. D., Stallone, M. C., & Mangano, S. (2018). A novel mutation of WDR62 gene associated with severe phenotype including infantile spasm, microcephaly, and intellectual disability. *Brain Dev, 40*(1), 58-64. doi:10.1016/j.braindev.2017.07.003

Naseer, M. I., Rasool, M., Abdulkareem, A. A., Chaudhary, A. G., Zaidi, S. K., & Al-Qahtani, M. H. (2019). Novel compound heterozygous mutations in WDR62 gene leading to developmental delay and Primary Microcephaly in Saudi Family. *Pak J Med Sci, 35*(3), 764-770. doi:10.12669/pjms.35.3.36

Naseer, M. I., Rasool, M., Sogaty, S., Chaudhary, R. A., Mansour, H. M., Chaudhary, A. G., . . . Al-Qahtani, M. H. (2017). A novel WDR62 mutation causes primary microcephaly in a large consanguineous Saudi family. *Ann Saudi Med, 37*(2), 148-153. doi:10.5144/0256-4947.2017.148

Nicholas, A. K., Khurshid, M., Desir, J., Carvalho, O. P., Cox, J. J., Thornton, G., . . . Woods, C. G. (2010). WDR62 is associated with the spindle pole and is mutated in human microcephaly. *Nature Genetics, 42*(11), 1010-U1138. doi:Doi 10.1038/Ng.682

Okamoto, N., Kohmoto, T., Naruto, T., Masuda, K., & Imoto, I. (2018). Primary microcephaly caused by novel compound heterozygous mutations in ASPM. *Hum Genome Var, 5*, 18015. doi:10.1038/hgv.2018.15

Pagnamenta, A. T., Howard, M. F., Knight, S. J., Keays, D. A., Quaghebeur, G., Taylor, J. C., & Kini, U. (2016). Activation of an exonic splice-donor site in exon 30 of CDK5RAP2 in a patient with severe microcephaly and pigmentary abnormalities. *Clin Case Rep, 4*(10), 952-956. doi:10.1002/ccr3.663

Pagnamenta, A. T., Murray, J. E., Yoon, G., Sadighi Akha, E., Harrison, V., Bicknell, L. S., . . . Knight, S. J. (2012). A novel nonsense CDK5RAP2 mutation in a Somali child with primary microcephaly and sensorineural hearing loss. *Am J Med Genet A, 158A*(10), 2577-2582. doi:10.1002/ajmg.a.35558

Papari, E., Bastami, M., Farhadi, A., Abedini, S. S., Hosseini, M., Bahman, I., . . . Najmabadi, H. (2013). Investigation of primary microcephaly in Bushehr province of Iran: novel STIL and ASPM mutations. *Clin Genet, 83*(5), 488-490. doi:10.1111/j.1399-0004.2012.01949.x

Perez, Y., Bar-Yaacov, R., Kadir, R., Wormser, O., Shelef, I., Birk, O. S., . . . Birnbaum, R. Y. (2019). Mutations in the microtubule-associated protein MAP11 (C7orf43) cause microcephaly in humans and zebrafish. *Brain, 142*(3), 574-585. doi:10.1093/brain/awz004

Poulton, C. J., Schot, R., Seufert, K., Lequin, M. H., Accogli, A., Annunzio, G. D., . . . Mancini, G. M. (2014). Severe presentation of WDR62 mutation: is there a role for modifying genetic factors? *Am J Med Genet A, 164A*(9), 2161-2171. doi:10.1002/ajmg.a.36611

Rupp, V., Rauf, S., Naveed, I., Windpassinger, C., & Mir, A. (2014). A novel single base pair duplication in WDR62 causes primary microcephaly. *Bmc Medical Genetics, 15*, 107. doi:10.1186/s12881-014-0107-4

Sajid Hussain, M., Marriam Bakhtiar, S., Farooq, M., Anjum, I., Janzen, E., Reza Toliat, M., . . . Hansen, L. (2013). Genetic heterogeneity in Pakistani microcephaly families. *Clin Genet, 83*(5), 446-451. doi:10.1111/j.1399-0004.2012.01932.x

Sgourdou, P., Mishra-Gorur, K., Saotome, I., Henagariu, O., Tuysuz, B., Campos, C., . . . Louvi, A. (2017). Disruptions in asymmetric centrosome inheritance and WDR62-Aurora kinase B interactions in primary microcephaly. *Sci Rep, 7*, 43708. doi:10.1038/srep43708

Shaheen, R., Maddirevula, S., Ewida, N., Alsahli, S., Abdel-Salam, G. M. H., Zaki, M. S., . . . Alkuraya, F. S. (2019). Genomic and phenotypic delineation of congenital microcephaly. *Genet Med, 21*(3), 545-552. doi:10.1038/s41436-018-0140-3

Shen, J., Eyaid, W., Mochida, G. H., Al-Moayyad, F., Bodell, A., Woods, C. G., & Walsh, C. A. (2005). ASPM mutations identified in patients with primary microcephaly and seizures. *J Med Genet, 42*(9), 725-729. doi:10.1136/jmg.2004.027706

Sukumaran, S. K., Stumpf, M., Salamon, S., Ahmad, I., Bhattacharya, K., Fischer, S., . . . Noegel, A. A. (2017). CDK5RAP2 interaction with components of the Hippo signaling pathway may play a role in primary microcephaly. *Mol Genet Genomics, 292*(2), 365-383. doi:10.1007/s00438-016-1277-x

Tan, C. A., Topper, S., Ward Melver, C., Stein, J., Reeder, A., Arndt, K., & Das, S. (2014). The first case of CDK5RAP2-related primary microcephaly in a non-consanguineous patient identified by next generation sequencing. *Brain Dev, 36*(4), 351-355. doi:10.1016/j.braindev.2013.05.001

Wang, R., Khan, A., Han, S., & Zhang, X. (2017). Molecular analysis of 23 Pakistani families with autosomal recessive primary microcephaly using targeted next-generation sequencing. *J Hum Genet, 62*(2), 299-304. doi:10.1038/jhg.2016.128

Yamamoto, S., Jaiswal, M., Charng, W. L., Gambin, T., Karaca, E., Mirzaa, G., . . . Bellen, H. J. (2014). A drosophila genetic resource of mutants to study mechanisms underlying human genetic diseases. *Cell, 159*(1), 200-214. doi:10.1016/j.cell.2014.09.002

Yang, Y. J., Baltus, A. E., Mathew, R. S., Murphy, E. A., Evrony, G. D., Gonzalez, D. M., . . . Walsh, C. A. (2012). Microcephaly gene links trithorax and REST/NRSF to control neural stem cell proliferation and differentiation. *Cell, 151*(5), 1097-1112. doi:10.1016/j.cell.2012.10.043

Yi, Y. G., Lee, D. W., Kim, J., Jang, J. H., Lee, S. M., & Jang, D. H. (2019). Two Novel Mutations (c.883-4_890del and c.1684C>G) of WDR62 Gene Associated With Autosomal Recessive Primary Microcephaly: A Case Report. *Front Pediatr, 7*, 457. doi:10.3389/fped.2019.00457

Yigit, G., Brown, K. E., Kayserili, H., Pohl, E., Caliebe, A., Zahnleiter, D., . . . Wollnik, B. (2015). Mutations in CDK5RAP2 cause Seckel syndrome. *Mol Genet Genomic Med, 3*(5), 467-480. doi:10.1002/mgg3.158

Yu, T. W., Mochida, G. H., Tischfield, D. J., Sgaier, S. K., Flores-Sarnat, L., Sergi, C. M., . . . Walsh, C. A. (2010). Mutations in WDR62, encoding a centrosome-associated protein, cause microcephaly with simplified gyri and abnormal cortical architecture. *Nat Genet, 42*(11), 1015-1020. doi:10.1038/ng.683

Zombor, M., Kalmar, T., Nagy, N., Berenyi, M., Telcs, B., Maroti, Z., . . . Sztriha, L. (2019). A novel WDR62 missense mutation in microcephaly with abnormal cortical architecture and review of the literature. *J Appl Genet, 60*(2), 151-162. doi:10.1007/s13353-019-00486-y
